# Recent advances in treating age-related macular degeneration and diabetic retinopathy: Current therapies and emerging novel approaches

**DOI:** 10.1007/s11357-025-01921-1

**Published:** 2025-10-12

**Authors:** Réka Antal, Zoltán Benyó, Zoltán Zsolt Nagy, Illés Kovács, János Tibor Fekete, Balázs Győrffy, Krisztián A. Kovács

**Affiliations:** 1 Translational Retina Research Group, Institute of Translational Medicine, Semmelweis University, Tűzoltó u. 37-47, 1094 Budapest, Hungary; 2 Institute of Translational Medicine, Semmelweis University, Tűzoltó u. 37-47, 1094 Budapest, Hungary; 3 HUN-REN-SU, Cerebrovascular and Neurocognitive Disease Research Group, Tűzoltó u. 37-47, 1094 Budapest, Hungary; 4 Department of Ophthalmology, Semmelweis University, Maria u. 39, Budapest, 1085 Hungary; 5 Department of Bioinformatics, Semmelweis University, Tűzoltó u. 7, 1094 Budapest, Hungary; 6 Cancer Biomarker Research Group, Institute of Molecular Life Sciences, HUN-REN Research Centre for Natural Sciences, 1117 Budapest, Hungary; 7 Department of Biophysics, Medical School, University of Pecs, 7624 Pecs, Hungary

**Keywords:** Neovascular age-related macular degeneration, Proliferative diabetic retinopathy, Vascular endothelial growth factor, Anti-VEGF, Gene therapy, Tyrosine kinase inhibitor

## Abstract

The incidence of retinopathies is expected to increase globally as our population ages and diabetes becomes more prevalent. Neovascular age-related macular degeneration (nAMD) and proliferative diabetic retinopathy (PDR) are major causes of severe vision loss. These ophthalmological conditions are associated with neovascularization caused by excessive vascular endothelial growth factor (VEGF) secretion. This review focuses on the milestones achieved during the last few decades in treating retinopathies, presents a meta-analysis of key human clinical trials, and finally describes various novel therapies, some of which could potentially eliminate the need for intravitreal injections of anti-VEGF in the future. According to data from clinical trials and everyday medical practice, treatments targeting VEGF are efficient; however, they can cause various systemic (such as stroke) and ocular adverse events. The most frequently experienced adverse reactions (such as endophthalmitis or retinal detachment) are related to the injection process itself. These complications warrant the efforts invested in developing novel approaches, including gene therapy-based products and inhibitors of VEGF receptors. We discuss here the strengths and drawbacks of gene therapy and highlight the potential of small-molecule tyrosine kinase inhibitors (TKIs) as alternative anti-neovascularization agents. TKIs have recently been tested via numerous administration routes, including simple oral dosing of oral formulations in addition to intravitreal implants, which allow for a slow release, intravitreal depots, and suprachoroidal injections. All of which have shown different safety and efficacy profiles. We conclude that gene therapy products, especially TKIs, have the potential to become inhibitors of ocular neovascularization in the upcoming years.

**Graphical Abstract:**

**Figure:**
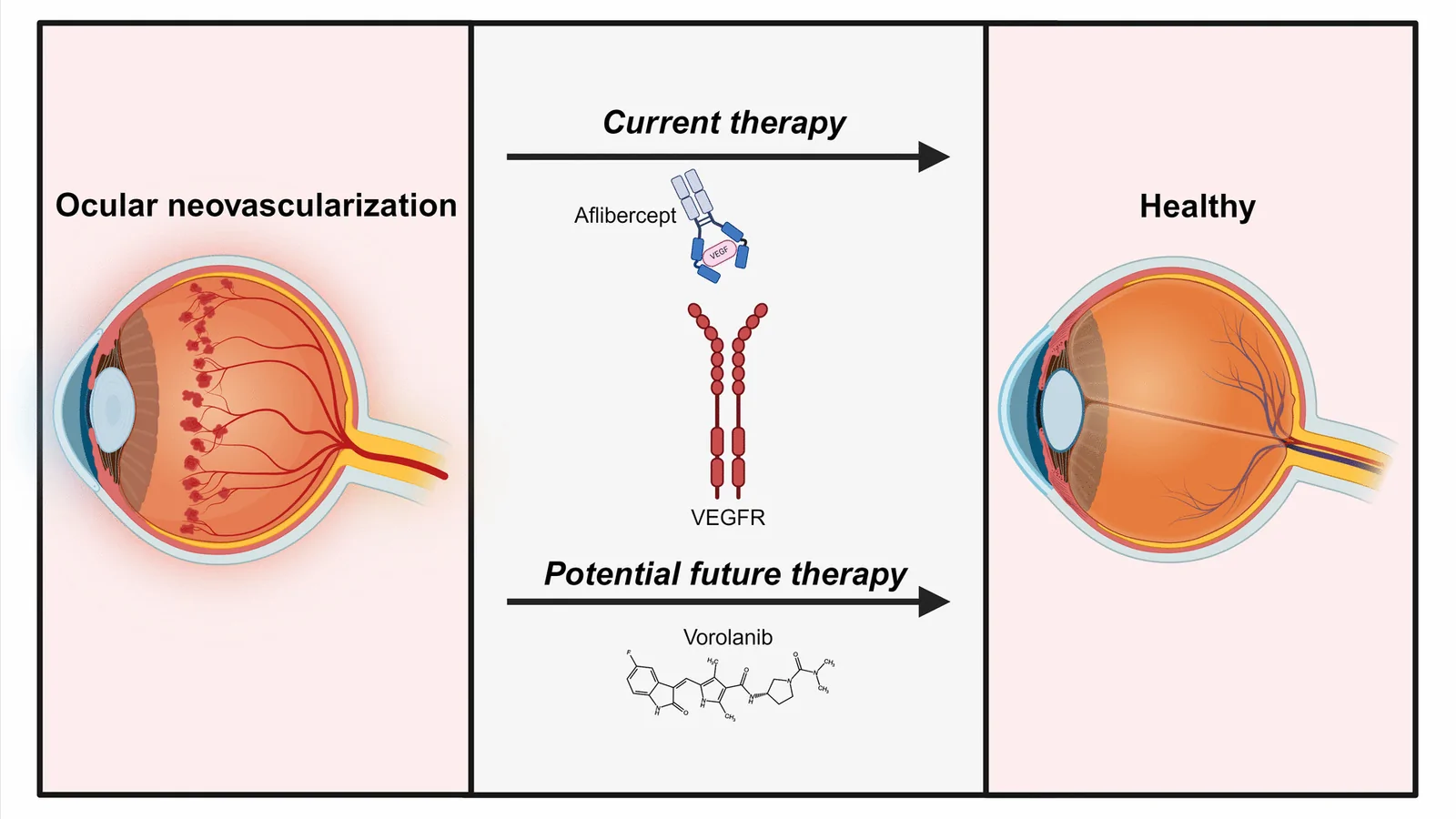

## Background

In 2020, the global prevalence of age-related macular degeneration (AMD) was estimated at 196 million, and the projected number of people with diabetic retinopathy (DR) in 2020/2021 was 103 million; therefore, AMD and DR are the leading causes of blindness in developed countries [1, 2]. The global AMD market size was valued at USD 9.8 billion in 2021 and is anticipated to witness a 6.9% compound annual growth rate (CAGR) from 2022 to 2030 [3]. The global DR market is predicted to reach USD 12 billion by 2025, growing at a CAGR of 6.9% during the forecast period (2019–2025) [4]. These ocular conditions and current therapeutic strategies constitute a huge financial burden for the social security services and the medical care system.

AMD has multifactorial pathogenesis. Genetic background and environmental stressors are both risk factors for the progression of this ocular disease. A key factor in the pathology of AMD is aging, which is associated with inflammaging, a low-grade, chronic inflammatory state, and with immunosenescence. These conditions have relevance to early AMD progression [5, 6]. Studies also suggest that mitochondrial damage in the aging retinal pigment epithelium (RPE) contributes to the pathology of AMD. There is a decrease in the function of the electron transport chain and an increase in the level of reactive oxygen species (ROS) in the aging retinal mitochondria [7, 8]. In the RPE of AMD patients, a reduction in the number of mitochondria and an increase in mtDNA damage were observed, which are probably caused by the elevated level of ROS. The mtDNA damage was associated with reduced ATP production, and the degree of RPE mtDNA damage correlated with the severity of AMD [9, 10]. Therefore, targeting RPE mitochondria could provide a therapeutic approach to treat early AMD. Furthermore, oxidative stress leads to inflammation, which contributes to the progression of neovascular AMD (nAMD), also termed wet AMD [11]. The enhanced migration of immune cells to the subretinal space and the upregulated production of pro-inflammatory cytokines play a huge role in choroidal neovascularization (CNV), a hallmark of nAMD. During CNV, which is a result of angiogenesis, new blood vessels form in the choroid and grow into the subretinal space [12, 13]. During this process, Bruch’s membrane is disrupted; thus, fluids and macromolecules can enter the neuroretina, and these events can lead to the loss of central visual acuity (VA) or even to permanent vision loss in people over the age of 50 [14].

Proliferative diabetic retinopathy (PDR) is a more advanced stage of DR, a major complication of diabetes mellitus, and is typically a consequence of a longer duration of diabetes, hyperglycemia, and hypertension. These factors can result in hypoxia, which contributes to neovascularization in the retina. Abnormal vessels may bleed, leading to hemorrhage in the vitreous, and can also trigger retinal detachment [15, 16]. Diabetic macular edema (DME) occurs when the leakage is located in the macular area. This condition is the primary cause of vision loss in the adult population and the elderly with diabetes.

**Fig. 1:**
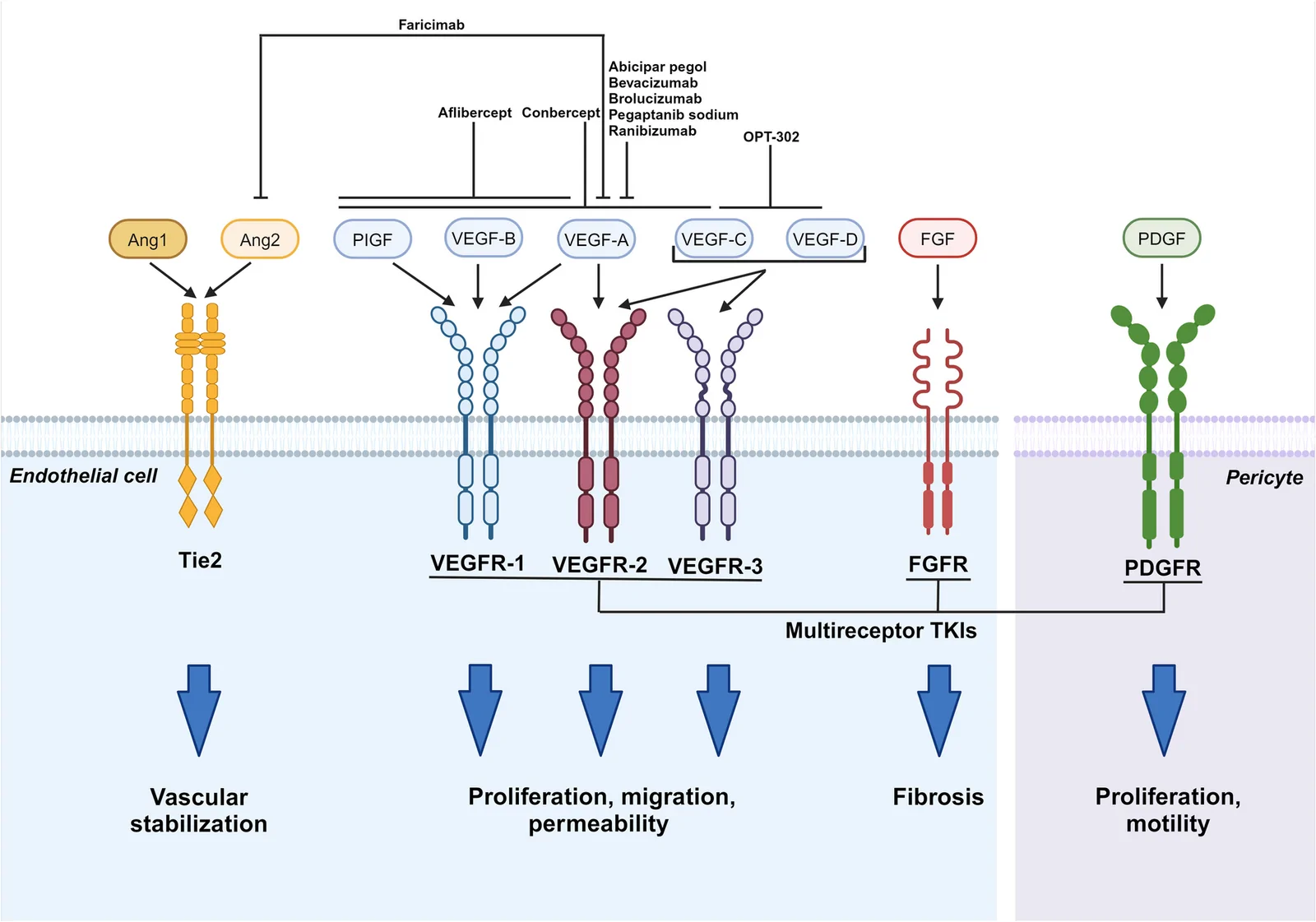
Summary of currently available anti-VEGF treatments and novel anti-angiogenic therapies for nAMD and PDR. Ang1, angiopoietin-1; Ang2, angiopoietin-2; FGF, fibroblast growth factor; FGFR, fibroblast growth factor receptor; PDGF, platelet-derived growth factor; PDGFR, platelet-derived growth factor receptor; PlGF, placental growth factor; Tie2, angiopoietin-1 and −2 receptor; TKIs, tyrosine kinase inhibitors; VEGF, vascular endothelial growth factor; VEGFR, vascular endothelial growth factor receptor. Created in BioRender.com

In both nAMD and PDR, neovascularization is associated with enhanced endothelial cell proliferation, migration, and an increase in vascular permeability as a consequence of excessive production of vascular endothelial growth factor (VEGF) by the RPE, glial cells (Müller cells and microglia), and macrophages [17, 18]. VEGF family members and their receptors play a crucial role in regulating various biological responses. VEGF-A, VEGF-B, VEGF-C, VEGF-D, and placental growth factor (PlGF) regulate embryogenesis, vasculogenesis, angiogenesis, and vascular homeostasis [19]. VEGF-A (also VEGF) interacts with two VEGF receptor (VEGFR) tyrosine kinases, VEGFR-1 and VEGFR-2 (also called KDR) (Fig. 1). Binding of VEGF to its receptors on endothelial cells promotes vasculogenesis, the de novo formation of a vascular network during embryogenesis, and angiogenesis, the development of new blood vessels from pre-existing ones [20, 21]. Interestingly, there is a splice variant, an alternative soluble form of VEGFR-1 that acts as a decoy receptor and negatively regulates VEGFR-2 by scavenging VEGF [22]. Although VEGF165, a splicing isoform of VEGF, on one hand, is a pro-angiogenic molecule and key regulator of physiological angiogenesis necessary for tissue regeneration and wound healing, on the other hand, it induces pathological angiogenesis in several diseases such as tumor neovascularization, rheumatoid arthritis, and ophthalmic conditions such as nAMD and PDR [23, 24]. VEGF-C and VEGF-D can also bind to VEGFR-2, regulating angiogenesis and vascular permeability, and are ligands for VEGFR-3 as well. The latter receptor is not only a regulator of lymphangiogenesis but is also involved in pathological angiogenesis (Fig. 1) [25]. Nakao et al. found that the expression of VEGFR-3 is upregulated in CNV lesions, mainly in macrophages and angiogenic endothelial cells; thus, it could play a role in the development of nAMD [26].

The present review article covers the pathogenesis of nAMD and PDR, focusing on the molecular aspects, provides a summary of the therapies currently used in standard medical practice, and discusses the benefits and drawbacks of novel approaches targeting VEGF/VEGFR signaling pathways. Moreover, a meta-analysis of earlier and the most recent human clinical trials is presented.

## Main text

### Methodology

Literature searches were conducted from inception to April 2024 and updated in December 2024 to identify relevant clinical studies focusing on anti-VEGF therapies in ocular neovascularization diseases. The electronic search was performed in MEDLINE, PubMed, Embase, and ClinicalTrials.gov. The search terms were (“age-related macular degeneration” OR “AMD” OR “neovascular AMD” OR “wet AMD” OR “proliferative diabetic retinopathy” OR “PDR” OR “diabetic macular edema” OR “DME”) AND (“anti-vascular endothelial growth factor” OR “anti-VEGF” OR “ranibizumab” OR “Lucentis” OR “aflibercept” OR “Eylea” OR “brolucizumab” OR “Beovu” OR “Abicipar pegol” OR “conbercept” OR “Lumitin”). Articles were screened by title and abstract, and the full text of eligible studies was analyzed if available. During the analysis, the following criteria were assessed: (1) study design, (2) condition (nAMD, PDR, or DME), (3) name of the anti-VEGF therapy, and (4) outcomes (BCVA and serious OAE (endophthalmitis)). All papers were initially searched by RA and assessed independently by RA and KAK.

This meta-analysis aimed to assess the efficacy of various anti-VEGF agents based on changes in patients’ BCVA from baseline and to analyze the risk of serious endophthalmitis occurring during anti-VEGF therapy. We included only randomized, controlled clinical trials that enrolled patients treated with intravitreal anti-VEGF agents. Only studies that compared anti-VEGF therapies (same regimen for a given anti-VEGF drug) with control agents (sham or non-anti-VEGF treatment) or with other anti-VEGF agents were included.

This frequentist network meta-analysis employed a random-effects framework using the inverse variance approach to estimate mean differences between treatment groups, with sham designated as the comparator. The performance of each treatment was evaluated using the P-score, a statistical measure that quantifies the relative ranking of interventions. In the analysis of SOAE (endophthalmitis), a continuity correction was applied to adjust for sparse data, ensuring robustness in effect size estimation. P-scores range from 0 to 1, where higher values denote a greater probability of a treatment being among the most effective, and lower values reflect a higher likelihood of a treatment being among the least effective [27]. To evaluate the coherence of the network, we examined discrepancies between direct and indirect comparisons, with significant inconsistency indicated by a low *p*-value. All analyses were carried out using the Netmeta package via the web platform www.metaanalysisonline.com [28].

### Initial approaches for the treatment of neovascularization

Laser photocoagulation has been the conventional treatment for DR and DME for almost three decades. In the 1970 s, a randomized clinical trial, the “Diabetic Retinopathy Study” (DRS), recruited patients with severe non-proliferative diabetic retinopathy (NPDR) or PDR. Panretinal photocoagulation (PRP) reduced the risk of severe visual loss by 50% to 60%. However, laser treatment caused some loss of the visual field, and eyes in the treated groups continued to show a VA decrease of one line [29]. According to the “Early Treatment Diabetic Retinopathy Study” (ETDRS) trial, which enrolled patients with DR in the 1980 s to investigate the efficacy and the optimal timing of laser photocoagulation in the treatment of DME, early treatment with PRP reduced the risk of developing high-risk PDR by 50% compared with the group of patients not accepting photocoagulation [30]. However, some serious ocular adverse events (SOAEs) occurred after laser photocoagulation treatment, including decreased VA, night blindness, loss of peripheral vision, and CNV [31]. Focal laser photocoagulation was a well-established method for treating nAMD patients with juxtafoveal and perifoveal CNV as well. Although there is a possibility of preventing further vision loss with subfoveal laser photocoagulation, it rarely improves VA, and there is a high recurrence rate after the treatment due to ocular adverse events (OAEs), including damage to the macula and retina, enlargement of laser scars, and development of secondary neovascularization [32–36]. According to studies investigating laser photocoagulation in patients with PDR and nAMD, the treatment caused several SOAEs; therefore, photocoagulation is considered a controversial treatment that is used much less frequently nowadays.

Although the gold standard treatment for CNV secondary to AMD was laser photocoagulation for several decades, the major limitations, low efficacy, and small number of patients eligible for the therapy have heightened the need for other types of treatment. In the 1990 s, there were some advances in the therapy of AMD with a photodynamic approach, based on molecules generating toxic active radicals upon targeted illumination of the fundus, specifically in neovessels. The “Treatment of AMD with Photodynamic Therapy” (TAP) Study Group investigated the efficacy of photodynamic therapy (PDT) with Verteporfin® (Visudyne, Novartis AG, Basel, Switzerland) in two phase III clinical trials [37–39]. Verteporfin is an established photosensitizer agent that must be administered by intravenous infusion; then, it is activated by nonthermal laser light in the 689–691 nm range. The excited photosensitizer drug generates singlet oxygen and other free radicals, thus inducing damage to the membranes of endothelial and blood cells. This process is followed by platelet adhesion and thrombosis of the neovasculature in the treated area, selectively destroying newly formed, undesired blood neovessels. According to the TAP study, 53% of verteporfin-treated patients lost fewer than 15 letters of VA. However, 47% of patients treated with verteporfin still lost ≥ 15 letters [38]. According to the investigation of the TAP Study Group, by month 36, several patients had clinically relevant adverse events (AEs), including injection site AEs (17.8%), infusion-related back pain (2.8%), and photosensitivity reaction (2.5%) [39]. Based on these results and observations, PDT can be efficient in some cases, and it is still recommended for CNV as a complement to anti-VEGF therapy; however, it is used less frequently in everyday medical practice also due to the decreased availability of verteporfin in Europe [40].

### Molecular/physiological background

It has been known since the 1990 s that VEGF, originally named vascular permeability factor (VPF), is a mediator of retinal neovascularization, and two main biological activities, angiogenesis and increased vascular permeability, correlate with ocular VEGF levels [41, 42]. Increased VEGF secretion in the eye results in pathological angiogenesis by enhancing endothelial cell migration and proliferation, and these can contribute to the appearance of nAMD and PDR [17].

**Fig. 2:**
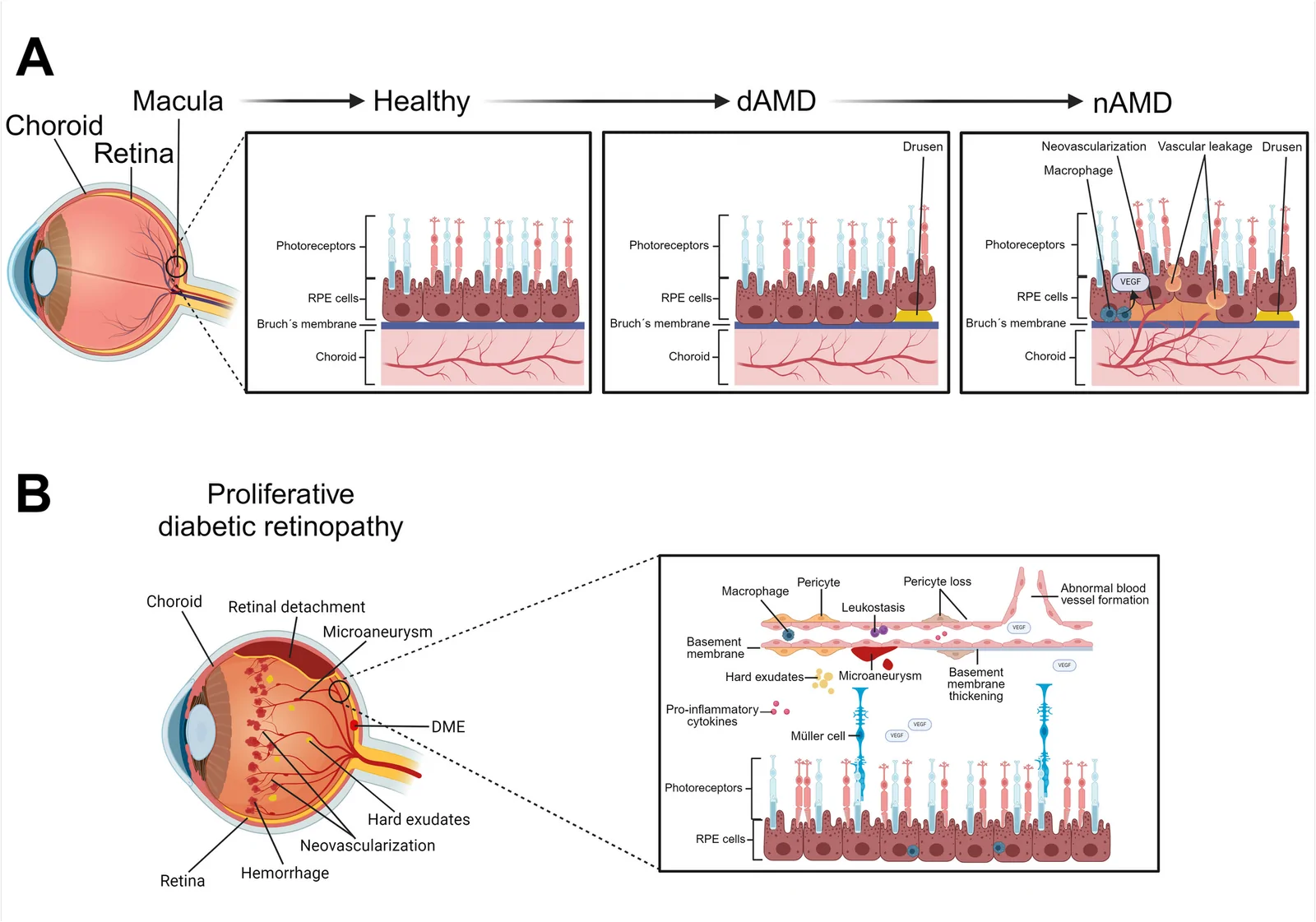
Main pathogenic events in age-related macular degeneration (AMD) and diabetic retinopathy (DR). **A** Representation of the main pathogenic events in the progression of AMD from healthy to neovascular age-related macular degeneration (nAMD). With retinal aging, the accumulation of drusen deposits can be observed between retinal pigment epithelium (RPE) cells and the choroid. We refer to this stage of AMD as dry AMD (dAMD). Vascular inflammation within the choroid and retina is another effect of aging. As a consequence of inflammation, macrophages migrate to the subretinal space, resulting in excessive vascular endothelial growth factor (VEGF) secretion. Upregulated VEGF production enhances the abnormal formation of blood vessels that disrupt Bruch’s membrane and causes vascular leakage into the retina. This stage of AMD is known as nAMD. **B** Main pathogenic events during DR progression. Non-proliferative diabetic retinopathy (NPDR) is characterized by basement membrane thickening and the loss of pericytes, resulting in microaneurysms and hemorrhages. Pro-inflammatory cytokine and VEGF production by the RPE, Müller cells, and macrophages are enhanced. Inflammation leads to leukostasis, the process of activation and adhesion of leukocytes to the endothelium. Hard exudates occur due to plasma leakage. When leakage occurs in the macula, diabetic macular edema (DME) progresses. Finally, PDR progression can be observed when hyperglycemia, hypoxia, and pro-inflammatory cytokine production contribute to VEGF upregulation, leading to neovascularization. Bleeding of abnormal blood vessels causes hemorrhages in the vitreous, which can result in retinal detachment. Created in BioRender.com

One of the reasons for upregulated VEGF production is vascular inflammation within the retina and choroid due to aging. As a result of inflammation, microglial cells migrate into the subretinal space, which, in turn, enhances pro-inflammatory cytokine production and excessive VEGF secretion. Upregulated VEGF production induces the growth of vessels originating from the choroid to the subretinal space (Fig. 2A). This process is ultimately responsible for nAMD progression [43].

DR is characterized by basement membrane thickening and pericyte loss, leading to the development of hemorrhages and microaneurysms. Pro-inflammatory cytokine and VEGF production by the RPE, Müller cells, and macrophages are upregulated [44]. Furthermore, advanced glycation in diabetic individuals can enhance the adherence of leukocytes to the retinal capillary endothelium, which plays a significant role in retinal capillary occlusion and the development of hypoxia, which is another major cause of VEGF upregulation [45]. This effect is mediated through the activation of hypoxia-inducible factor 1α (HIF-1α) that positively regulates VEGF transcription. Finally, the increased amount of VEGF leads to abnormal blood vessel formation in the vitreous. Bleeding of these fragile neovessels results in hemorrhages, which process, together with the neovessels anchored into the vitreous, can trigger tractional retinal detachment (Fig. 2B) [46].

Many of the anti-VEGF agents have been primarily developed to inhibit tumor angiogenesis; however, it is not surprising that after observing the key role of VEGF in neovascularization, its inhibition in ocular neovascular diseases has also emerged. Currently, intravitreal anti-VEGF therapy is the first-line treatment for nAMD and DME secondary to DR; thus, the market share of anti-VEGF drugs reaches an unprecedented value not only in the USA but globally as well. The global anti-VEGF therapeutics market size was estimated to be USD 22.7 billion in 2022, and it is expected to reach USD 34.9 billion by 2033, growing at a CAGR of 3.9% during the forecast period (2023–2033) [47].

### VEGF-Targeting biotechnological products

According to a recent analysis, the global anti-VEGF therapeutics market in 2022 was dominated by Eylea (aflibercept), followed by Lucentis (ranibizumab) and Beovu (brolucizumab) [48]. Lumitin (conbercept) and Vabysmo (faricimab) are further anti-VEGF agents of choice. Over the last 25 years, many clinical trials were conducted to study anti-VEGF therapeutics in ophthalmological indications, which are summarized in Table 1 and Table 2.

**Table 1:** Summary of key clinical trials on visual outcomes and occurring serious adverse events following intravitreal anti-vascular endothelial growth factor A treatment for age-related macular degeneration and diabetic retinopathy

Therapeutic modality	Approved indications	Study ref	Study design	No. of patients	Group	BCVA (ETDRS letters)		SOAEs^c^	SSAEs^d^	Trial registration number on clinicaltrials.gov^e^	Status
						Baseline	Change				
Pegaptanib sodium	(n)AMD	VISION [49]	Phase II/III	Total: 1190	Pegaptanib 0.3 mg	52.8 (12.6)^a^	− 7.5 (N.A.)^a^	Endophthalmitis (1.3%)		NCT00321997	Completed
				Control: 298	Pegaptanib 1.0 mg	50.7 (12.8)^a^	− 6.5 (N.A.)^a^				
					Pegaptanib 3.0 mg	51.1 (12.9)^a^	− 10.0 (N.A.)^a^				
					Sham	52.7 (13.0)^a^	− 15.0 (N.A.)^a^				
		MDRS [50]	Phase II	Total: 172	Pegaptanib 0.3 mg	57.1 (11.5)^a^	4.7 (N.A.)^a^			NCT00040313	Completed
				Control: 42	Pegaptanib 1.0 mg	55.0 (10.5)^a^	4.7 (N.A.)^a^				
					Pegaptanib 3.0 mg	57.0 (9.1)^a^	1.1 (N.A.)^a^				
					Sham	55.8 (9.5)^a^	− 0.4 (N.A.)^a^				
		LEVEL [51]	Phase IV	Total: 568	Pegaptanib 0.3 mg	49.6 (21.9)^a^	12.2 (18.9)^a^			NCT00354445	Unknown status
Ranibizumab	nAMD, DR, DME, macular edema following retinal vein occlusion, myopic CNV	MARINA [53]	Phase III	Total: 716	Ranibizumab 0.3 mg	53.1 (12.9)^a^	5.4 (N.A.)^a^	Uveitis (1.3%)		NCT00056836	Completed
				Control: 238	Ranibizumab 0.5 mg	53.7 (12.8)^a^	6.6 (N.A.)^a^	Presumed endophthalmitis (1.3%), uveitis (1.3%)			
					Sham	53.6 (14.1)^a^	− 14.9 (N.A.)^a^				
		ANCHOR [40]	Phase III	Total: 423	Ranibizumab 0.3 mg	47.0 (13.1)^a^	8.5 (N.A.)^a^			NCT00061594	Completed
				Control: 143	Ranibizumab 0.5 mg	47.1 (13.2)^a^	11.3(N.A.)^a^	Endophthalmitis (1.4%)			
					Verteporfin	45.5 (13.1)^a^	− 9.5 (N.A.)^a^				
		PIER [54]	Phase IIIb	Total: 184	Ranibizumab 0.3 mg	55.8 (12.2)^a^	− 1.6 (N.A.)^a^	Macular edema (1.7%), ocular hemorrhage (3.4%)		NCT00090623	Completed
				Control: 63	Ranibizumab 0.5 mg	53.7 (15.5)^a^	− 0.2 (N.A.)^a^				
					Sham	55.1 (13.9)^a^	− 16.3 (N.A.)^a^	Macular edema (3.2%), ocular hemorrhage (3.2%)			
		READ-2 [154]	Phase II	Total: 126	Ranibizumab 0.5 mg	N.A	7.7 (N.A.)			NCT00407381	Completed
				Control: 42	Laser	N.A	5.1 (N.A.)				
					Ranibizumab 0.5 mg + laser	N.A	6.8 (N.A.)				
		RESTORE [55]	Phase III	Total: 345	Ranibizumab 0.5 mg	64.8 (10.1)	6.1 (6.4)			NCT00687804	Completed
				Control 111	Ranibizumab 0.5 mg + Laser	63.4 (10.0)	5.9 (7.9)	Cataract (2.5%)			
					Laser	62.4 (11.1)	0.8 (8.6)				
		CATT [58]	Phase III	Total: 1107	Ranibizumab 0.5 mg monthly	59.9 (14.2)^a^	8.8 (15.9)^a^		Death, all causes (5.3%)	NCT00593450	Completed
					Ranibizumab 0.5 mg PRN	61.6 (13.1)^a^	6.7 (14.6)^a^				
		RISE [56]	Phase III	Total: 377	Ranibizumab 0.3 mg	54.7 (12.6)	12.5 (14.1)		Cardiac failure congestive (5.6%), coronary artery disease (4.8%), myocardial infarction (4.8%)	NCT00473330	Completed
				Control: 127	Ranibizumab 0.5 mg	56.9 (11.6)	11.9 (12.1)	Vitreous hemorrhage (2.4%)	Cardiac failure congestive (5.6%)		
					Sham + ranibizumab 0.5 mg	57.2 (11.1)	2.6 (13.9)	Vitreous hemorrhage (4%)			
		RIDE [56]	Phase III	Total: 382	Ranibizumab 0.3 mg	57.5 (11.6)	10.9 (10.4)	Endophthalmitis (2.4%)	Myocardial infarction (6.4%)	NCT00473382	Completed
				Control: 130	Ranibizumab 0.5 mg	56.9 (11.8)	12.0 (14.9)	Cataract (2.4%), VA reduced (1.6%), endophthalmitis (1.6%)	Coronary artery disease (4.8%), pneumonia (6.5%)		
					Sham	57.3 (11.2)	2.3 (14.2)	VA reduced (1.6%), vitreous hemorrhage (2.4%)	Cardiac failure congestive (4.7%), myocardial infarction (4.7%), pneumonia (4%)		
		LUCIDATE [57]	Phase IV	Total: 33	Ranibizumab 0.5 mg	70.4 (4.9)	6.0 (8.5)		N.A	NCT01223612	Completed
				Control: 11	Laser	63.8 (5.7)	− 0.9 (10.6)		N.A		
		PROTOCOL T [162]	Phase III	Total: 666	Ranibizumab 0.3 mg	64.8 (11.3)^a^	8.3 (6.8)^a^		Renal failure (4.1%)	NCT01627249	Completed
Bevacizumab	Metastatic colorectal cancer, non-squamous non-small lung cancer, glioblastoma, cervical cancer	IBENA [163]	Phase I	Total: 45	Bevacizumab 1.0 mg	1.19 (0.23)^b^	− 0.03 (0.17)^b^			N.A	Completed
					Bevacizumab 1.5 mg	1.11 (0.36)^b^	− 0.06 (0.09)^b^				
					Bevacizumab 2.0 mg	1.35 (0.19)^b^	− 0.10 (0.10)^b^				
		IBEPE [164]	Phase I	Total: 12	Bevacizumab 1.5 mg	0.90 (0.11)^b^	− 0.14 (0.12)^b^			N.A	Completed
		ABC [165]	Phase III	Total: 131	Bevacizumab 1.25 mg	N.A	7.0 (N.A.)^a^	Uveitis (3%), vitreous hemorrhage (2%)	N.A	2006–001544-31^e^	Completed
				Control: 66	Standard therapy (pegaptanib/verteporfin/sham)	N.A	− 9.4 (N.A.)^a^	Uveitis (2%), rhegmatogenous retinal detachment (2%)	N.A		
		CATT [58]	Phase III	Total: 1107	Bevacizumab 1.25 mg monthly	60.2 (13.6)^a^	7.8 (15.5)^a^		Death, all causes (6.1%)	NCT00593450	Completed
					Bevacizumab 1.25 mg PRN	60.6 (13.0)^a^	5.0 (17.9)^a^				
		BOLT [166]	Phase N.A	Total: 65	Bevacizumab 1.25 mg	55.8 (9.7)	8.6 (9.1)	Increased intraocular pressure (2.4%)	Myocardial infarction (4.8%)	2007–000847-89^e^	Completed
				Control: 28	Laser	55.4 (7.9)	− 0.5 (10.6)	VA reduced (2.6%), vitreomacular traction with macular edema (2.6%)			
		PROTOCOL T [162]	Phase III	Total: 666	Bevacizumab 1.25 mg	64.8 (11.3)^a^	7.5 (7.4)^a^			NCT01627249	Completed
		CADME [167]	Phase II	Total: 62	Bevacizumab 1.25 mg	64.0 (N.A.)	5.3 (N.A.)	N.A	N.A	NCT01610557	Completed
					Ranibizumab 0.3 mg		6.6 (N.A.)				
		NCT03668054 [168]	Phase III	Total: 22	Bevacizumab 1.25 mg	53.0 (N.A.)^a^	7.0 (N.A.)^a^			NCT03668054	Completed
Brolucizumab	nAMD, DME	OSPREY [66]	Phase II	Total: 89	Brolucizumab 6.0 mg	54.1 (13.9)	5.8 (12.7)	Retinal tear (2.3%), retinal detachment (2.3%)		NCT01796964	Completed
				Control: 45	Aflibercept 2.0 mg	55.6 (12.3)	6.9 (9.3)		Atrial fibrillation (4.4%)		
		HAWK [67]	Phase III	Total: 1078	Brolucizumab 3.0 mg	61.0 (13.6)	5.6 (0.8)			NCT02307682	Completed
				Control: 360	Brolucizumab 6.0 mg	60.8 (13.7)	5.9 (0.8)				
					Aflibercept 2.0 mg	60.0 (13.9)	5.3 (0.8)				
		HARRIER [67]	Phase III	Total: 739	Brolucizumab 6.0 mg	61.5 (12.6)	6.1 (0.7)			NCT02434328	Completed
				Control: 369	Aflibercept 2.0 mg	60.8 (12.9)	6.6 (0.7)				
		MERLIN [68]	Phase IIIa	Total: 535	Brolucizumab 6.0 mg	73.4 (7.4)	0.3 (9.0)	Uveitis (1.1%)		NCT03710564	Terminated
				Control: 179	Aflibercept 2.0 mg	73.8 (7.2)	0.9 (6.5)				
		KITE [151]	Phase III	Total: 360	Brolucizumab 6.0 mg	66.0 (10.8)	10.6 (N.A.)	Endophthalmitis (1.1%)		NCT03481660	Completed
				Control: 181	Aflibercept 2.0 mg	63.7 (11.7)	9.4 (N.A.)				
		KESTREL [151]	Phase III	Total: 566	Brolucizumab 3.0 mg	65.7 (11.1)	7.3 (N.A.)	Retinal vasculitis (1.1%), endophthalmitis (1.1%)		NCT03481634	Completed
				Control: 190	Brolucizumab 6.0 mg	66.6 (9.7)	9.2 (N.A.)	Cataract (2.7%)			
					Aflibercept 2.0 mg	65.2 (12.4)	10.5 (N.A.)	Cataract (1.6%)			
		KINGFISHER [69]	Phase III	Total: 517	Brolucizumab 6.0 mg	61.3 (10.1)	12.2 (N.A.)			NCT03917472	Completed
				Control: 171	Aflibercept 2.0 mg	60.5 (11.3)	11.0 (N.A.)				
Abicipar pegol	None	REACH [169]	Phase II	Total: 64	Abicipar 1.0 mg	58.4 (13.5)	6.3 (7.8)			NCT01397409	Completed
				Control: 16	Abicipar 2.0 mg	58.5 (14.3)	8.2 (7.9)				
					Ranibizumab 0.5 mg	60.4 (16.4)	5.3 (11.1)				
		SEQUOIA [73]	Phase III	Total: 949	Abicipar 2q8	57.8 (12.1)	8.3 (14.3)	Retinal detachment (1%), uveitis (2.2%), retinal vasculitis (1.3%), vitritis (1.3%), endophthalmitis (1.6%)		NCT02462486	Completed
				Control: 318	Abicipar 2q12	56.3 (12.5)	7.3 (13.8)	Cataract (1%), uveitis (1.3%), retinal vasculitis (1.3%), VA reduced (1%), endophthalmitis (1.3%)			
					Ranibizumab 0.5q4	57.0 (12.3)	8.3 (11.8)	Retinal hemorrhage (1.6%)			
		CEDAR [73]	Phase III	Total: 939	Abicipar 2q8	56.7 (13.3)	6.7 (12.9)	Retinal hemorrhage (1.3%), uveitis (3.2%), retinal vasculitis (1.9%), VA reduced (1.3%), vitritis (1.6%), cataract (1%), retinal detachment (1%)		NCT02462928	Completed
				Control: 312	Abicipar 2q12	56.3 (13.1)	5.6 (13.3)	Retinal artery occlusion (1%), uveitis (3.2%), iridocyclitis (1.3%), retinal vasculitis (1%), VA reduced (1%), endophthalmitis (1.3%)			
					Ranibizumab 0.5q4	56.5 (12.6)	8.5 (13.6)		Pneumonia (4.5%)		
		MAPLE [74]	Phase II	Total: 122	Abicipar 2.0 mg	62.0 (12.19)	3.6 (7.2)			NCT03539549	Completed

Data are expressed as mean (standard deviation). *BCVA*, best-corrected visual acuity; *ETDRS*, Early Treatment of Diabetic Retinopathy Study; *SOAEs*, serious ocular adverse events; *SSAEs*, serious systemic adverse events; *PRN*, pro re nata; *2q8*, 2 mg every 8 weeks; *2q12*, 2 mg every 12 weeks; *0.5q4*, 0.5 mg every 4 weeks; *N.A.*, not available. ^a^Visual acuity. ^b^LogMAR. ^c^SOAEs occurring in ≥ 1% of patients in study eye. ^d^SSAEs occurring in ≥ 3% of patients. ^e^Trial registration number on clinicaltrialsregister.eu

**Table 2:** Summary of key clinical trials on visual outcomes and occurring serious adverse events following intravitreal anti-vascular endothelial growth factor A, -B, -C, -D, anti-placental growth factor, and anti-angiopoietin-2 treatment for age-related macular degeneration and diabetic retinopathy

Therapeutic modality	Approved indications	Study ref	Study design	No. of patients	Group	BCVA (ETDRS letters)		SOAEs^a^	SSAEs^b^	Trial registration number on clinicaltrials.gov	Status
						Baseline	Change				
Aflibercept	nAMD, macular edema following retinal vein occlusion, DME, myopic CNV, metastatic colorectal cancer	VIEW 1 [61]	Phase III	Total: 1215	Aflibercept 0.5q4	55.6 (13.1)	6.9 (13.4)			NCT00509795	Completed
				Control: 304	Aflibercept 2q4	55.2 (13.2)	10.9 (13.8)		Fall (4.3%)		
					Aflibercept 2q8	55.7 (12.8)	7.9 (15.0)		Fall (5.3%)		
					Ranibizumab 0.5q4	54.0 (13.4)	8.1 (15.3)		Pneumonia (4.6%)		
		VIEW 2 [61]	Phase III	Total: 1202	Aflibercept 0.5q4	51.6 (14.2)	9.7 (14.1)	Cataract (1.4%), retinal hemorrhage (1.4%)		NCT00637377	Completed
				Control: 291	Aflibercept 2q4	52.8 (13.9)	7.6 (12.6)	Cataract (1.3%), retinal hemorrhage (1%), AMD (1%)			
					Aflibercept 2q8	51.6 (13.9)	8.9 (14.4)	Cataract (1.3%), macular degeneration (1%), VA reduced (2.3%)			
					Ranibizumab 0.5q4	53.8 (13.5)	9.4 (13.5)	Cataract (1.8%), retinal detachment (1%), retinal hemorrhage (1.4%), VA reduced (1%)			
		TURF [62]	Phase IV	Total: 46	Aflibercept 2.0 mg	74.2 (N.A.)	0.2 (N.A.)		Urinary tract infection (4.5%), squamous cell carcinoma (4.5%), upper respiratory tract infection (11.1%)	NCT01543568	Completed
		ARIES [63]	Phase IIIb/IV	Total: 271	Aflibercept 2.0 mg early-T&E	60.2 (12.1)	4.3 (13.4)			NCT02581891	Completed
					Aflibercept 2.0 mg late-T&E	61.3 (10.8)	7.9 (11.9)	VA reduced (1.5%)	Pneumonia (3.7%)		
		PROTOCOL T [162]	Phase III	Total: 666	Aflibercept 2.0 mg	64.8 (11.3)^c^	8.0 (7.6)^c^			NCT01627249	Completed
		VISTA [64]	Phase III	Total: 461	Aflibercept 2q4	58.9 (10.8)	10.4 (14.2)	Cataract (2.6%), retinal detachment (2%), retinal vascular disorder (1.3%), vitreous hemorrhage (2%)	Anemia (6.5%), cardiac failure congestive (7.1%), coronary artery disease (3.2%), cellulitis (5.8%), osteomyelitis (4.5%), pneumonia (3.9%), hypoglycemia (3.9%), osteoarthritis (3.2%), cerebrovascular accident (3.2%), renal failure (3.2%), renal failure acute (6.5%), renal failure chronic (3.2%), hypertension (3.9%)	NCT01363440	Completed
				Control: 154	Aflibercept 2q8	59.4 (10.9)	10.5 (12.7)	Vitreous hemorrhage (1.3%)	anemia (4%), cardiac failure congestive (4%), coronary artery stenosis (3.3%), cellulitis (5.3%), fall (3.3%)		
					Laser	59.7 (10.9)	1.4 (14.5)	Diabetic retinopathy (1.3%), vitreous hemorrhage (2%)	Cardiac failure congestive (3.9%), cellulitis (3.3%), osteomyelitis (3.3%), hyperkalemia (3.9%), renal failure (3.3%), renal failure acute (5.8%)		
		VIVID [64]	Phase III	Total: 404	Aflibercept 2q4	60.8 (10.7)	10.3 (12.5)	Cataract (3.7%), vitreous hemorrhage (2.9%), cataract operation (2.2%)		NCT01331681	Completed
				Control: 133	Aflibercept 2q8	58.8 (11.2)	11.7 (10.1)	Cataract (5.2%), cataract subcapsular (1.5%), retinal detachment (1.5%)			
					Laser	60.8 (10.6)	1.6 (12.7)	Cataract (1.5%), diabetic retinopathy (1.5%), retinal neovascularization (2.3%), vitreous hemorrhage (1.5%)			
Faricimab	nAMD, DME	BOULEVARD [152]	Phase II	Total: 166	Faricimab 1.5 mg	60.9 (11.1)	11.7 (N.A.)	VA reduced (1.8%)	Pneumonia (3.6%)	NCT02699450	Completed
				Control: 59	Faricimab 6.0 mg	60.0 (11.0)	13.9 (N.A.)	Retinal vein occlusion (1.3%), vitreous hemorrhage (1.3%)			
					Ranibizumab 0.3 mg	61.2 (9.9)	10.3 (N.A.)	Diabetic retinopathy (1.2%)			
		STAIRWAY [153]	Phase II	Total: 71	Faricimab 6q12	57.8 (10.5)	10.1 (N.A.)		Acute left ventricular failure (4.2%), coronary artery disease (4.2%), vertigo (4.2%), fall (4.2%), headache (4.2%), ischemic stroke (4.2%), mental status changes (4.2%)	NCT03038880	Completed
				Control: 16	Faricimab 6q16	60.4 (10.8)	11.4 (N.A.)		Atrial fibrillation (3.2%), sepsis (3.2%), metastatic neoplasm (3.2%)		
					Ranibizumab 0.5q4	55.3 (12.1)	9.6 (N.A.)				
		TENAYA [71]	Phase III	Total: 671	Faricimab 6q16	61.3 (12.5)	5.8 (N.A.)			NCT03823287	Completed
				Control: 337	Aflibercept 2q8	61.5 (12.9)	5.1 (N.A.)	Cataract (1.5%), nAMD (2.7%)			
		LUCERNE [71]	Phase III	Total: 658	Faricimab 6q16	58.7 (14.0)	6.6 (N.A.)	nAMD (2.4%)		NCT03823300	Completed
				Control: 327	Aflibercept 2q8	58.9 (13.3)	6.6 (N.A.)	nAMD (1.2%)			
		YOSEMITE [72]	Phase III	Total: 940	Faricimab 6q8	62.0 (9.9)	10.7 (N.A.)	Cataract (1.3%), DR (1.3%), vitreous hemorrhage (1%)		NCT03622580	Completed
				Control: 312	Faricimab 6.0 mg T&E	61.9 (10.2)	10.7 (N.A.)	Cataract (1%), uveitis (1.3%), endophthalmitis (1.3%)			
					Aflibercept 2q8	62.2 (9.5)	11.4 (N.A.)	Cataract (1%)			
		RHINE [72]	Phase III	Total: 951	Faricimab 6q8	62.5 (10.1)	10.9 (N.A.)	Cataract (1.3%), diabetic retinal edema (2.2%), vitreous hemorrhage (1.3%)		NCT03622593	Completed
				Control: 315	Faricimab 6.0 mg T&E	61.3 (10.3)	10.1 (N.A.)	Cataract (2.2%)			
					Aflibercept 2q8	62.5 (10.0)	9.4 (N.A.)	Cataract (2.2%), DR (1%)			
Conbercept	nAMD (only in China)	AURORA [170]	Phase II	Total: 105	Conbercept 0.5 mg PRN	46.6 (14.5)	14.3 (17.1)	VA reduced (3.4%), cataract progression (1.7%)		NCT01157715	Completed
					Conbercept 0.5 mg monthly	50.8 (12.9)	9.3 (11.0)				
					Conbercept 2.0 mg PRN	47.6 (13.7)	12.4 (16.4)	Endophthalmitis (1.6%)			
					Conbercept 2.0 mg monthly	48.9 (14.7)	15.4 (14.7)				
		PHOENIX [75]	Phase III	Total: 124	Conbercept 0.5 mg	49.0 (17.1)	10.0 (14.0)	Cataract (1.2%)		NCT01436864	Completed
				Control: 43	Sham	48.0 (14.1)	8.8 (13.9)	Retinal detachment (2.4%)			
		SAILING [76]	Phase III	Total: 249	Conbercept 0.5 mg	56.6 (11.5)	8.2 (9.5)	N.A	N.A	NCT02194634	Unknown status
				Control: 124	Laser	57.6 (11.5)	0.3 (12.0)	N.A	N.A		
OPT-302	None	NCT03345082 [77]	Phase IIb	Total: 366	OPT-302 0.5 mg + ranibizumab 0.5 mg	51.1 (9.0)	9.4 (11.3)	Endophthalmitis (0.8%), vitritis (0.8%)	Cardiac disorders (3.3%), gastrointestinal disorders (3.3%)	NCT03345082	Completed
				Control: 121	OPT-302 2.0 mg + ranibizumab 0.5 mg	49.5 (10.3)	14.2 (11.6)		Cardiac disorders (3.2%)		
					Sham + ranibizumab 0.5 mg	50.7 (10.2)	10.8 (11.5)		Infections and infestations (3.3%)		
		NCT03397264 [78]	Phase IIa	Total: 153	OPT-302 2.0 mg + aflibercept 2.0 mg	N.A	5.9 (0.8)			NCT03397264	Completed
				Control: 48	Sham + aflibercept 2.0 mg	N.A	6.1 (1.0)				

Data are expressed as mean (standard deviation). *BCVA*, best-corrected visual acuity; *ETDRS*, Early Treatment of Diabetic Retinopathy Study; *SOAEs*, serious ocular adverse events; *SSAEs*, serious systemic adverse events; *0.5q4*, 0.5 mg every 4 weeks; *2q4*, 2 mg every 4 weeks; *2q8*, 2 mg every 8 weeks; *T&E*, treat-and-extend; *6q12*, 6 mg every 12 weeks; *6q16*, 6 mg every 16 weeks; *6q8*, 6 mg every 8 weeks; *PTI*, personalized treatment interval; *N.A.*, not available. ^a^SOAEs occurring in ≥ 1% of patients in study eye. ^b^SSAEs occurring in ≥ 3% of patients. ^c^Visual acuity

#### Pegaptanib sodium

Pegaptanib sodium (Macugen®; Eyetech Pharmaceuticals, New York, NY, USA) was one of the earliest agents developed to inhibit VEGF. It is a ribonucleic acid aptamer that selectively binds to its target molecule with high affinity and therefore blocks the action of VEGF-165 (Fig. 1), the main regulator of neovascularization and vascular permeability.

Pegaptanib was one of the first ocular pharmacotherapies to undergo clinical trials for the treatment of both nAMD and PDR. In the VISION trial, 70% of patients with nAMD who received 0.3 mg pegaptanib lost fewer than 15 letters of VA compared with 55% of patients in the control group. The risk of losing ≥ 15 letters was reduced by 33% at the end of the first year (Table 1) [49]. According to published data from a phase IV, open-label, uncontrolled study (LEVEL), during the first year, VA improved by a mean of 12.2 ± 18.9 letters in the 0.3 mg pegaptanib group (Table 1) [50, 51]. As a result, pegaptanib was the first FDA-approved intravitreal treatment for nAMD in 2004 that is based on the inhibition of VEGF; however, it is not approved by the FDA for the treatment of DME.

#### Ranibizumab

Ranibizumab (Lucentis™; Genentech, San Francisco, CA, USA) is a monoclonal antibody fragment that binds to all VEGF-A isoforms and inhibits their action (Fig. 1) [52].

Three phase III studies have investigated the use of intravitreal injections of ranibizumab in patients with nAMD. At the end of the second year, the mean increase in VA was 6.6 letters in the 0.5 mg ranibizumab group in the MARINA study, while during the first year, patients treated with monthly ranibizumab injections (0.5 mg group) in the ANCHOR study gained a mean of 11.3 letters of VA (Table 1) [40, 53]. However, the PIER study yielded completely different results. During the first year, there was a mean loss of 0.2 letters in the 0.5 mg ranibizumab group (Table 1) [54]. Later in the 2010 s, three phase III studies (RESTORE, RISE, and RIDE) [55, 56] and a phase IV study (LUCIDATE) [57] were performed to test the effectiveness of 0.5 mg ranibizumab intravitreal injections in PDR patients (Table 1). Currently, ranibizumab is approved by the FDA and EMA for the treatment of both nAMD and DR.

#### Bevacizumab

Bevacizumab (Avastin®; Genentech, San Francisco, CA, USA) is a full-length humanized monoclonal antibody. Similarly to ranibizumab, it binds all isoforms of VEGF-A (Fig. 1). It was first approved by the FDA as a first-line treatment for metastatic colorectal cancer, and since then, it has been licensed for many other cancer types. Despite its efficacy in ocular conditions with a neovascularization-based pathology, it is still used only in an off-label manner for CNV secondary to nAMD and DME related to PDR.

Due to the serious and non-serious AEs observed in each clinical trial, numerous concerns were raised with intravitreal injections of bevacizumab and other monoclonal antibodies, like ranibizumab, including the risk of causing intraocular inflammation (IOI). The “Comparison of Age-related Macular Degeneration Treatments Trials” (CATT) study reported endophthalmitis in 1% and 0.7% of patients treated monthly with bevacizumab and ranibizumab injections pro re nata, respectively (Table 1) [58]. The fact that intravitreally administered monoclonal antibodies can be found in the systemic circulation as well, where they can target and block VEGF-A, also requires attention [59]. As a result, the risk of systemic thromboembolic AEs also increased in the CATT study. The rates of arteriothrombotic events were 5% in the bevacizumab-treated group and 4.7% in the ranibizumab-treated group [58]. The CATT study has been initiated by the National Eye Institute (USA) to refute arguments against the ophthalmological applications of bevacizumab that cost approximately 250 lower than ranibizumab and to support the more cost-effective treatment of patients suffering from ocular neovascularization. However, the bevacizumab formulation intended for intravenous infusion has to be re-dispensed that can compromise sterility. Furthermore, bevacizumab is a larger molecule (149 kDa) than ranibizumab (48 kDa) and may, therefore, when administered intravitreally, be detected longer in the systemic circulation than ranibizumab. The latter may leave the eye and be cleared from the systemic circulation faster [60].

#### Aflibercept

Aflibercept (Eylea®; Bayer HealthCare, Whippany, NJ, USA) is another anti-VEGF drug licensed for the treatment of nAMD and DR. This molecule, also called as VEGF-Trap, is a recombinant fusion protein consisting of the extracellular immunoglobulin (Ig) domains of VEGFR-1 and VEGFR-2 fused to the Fc region of IgG1; thus, aflibercept can bind to VEGF-A, VEGF-B, and PlGF (Fig. 1).

Three clinical trials (VIEW 1, VIEW 2, TURF) [61, 62] were conducted to test the efficacy and safety of intravitreal injection of 2 mg aflibercept in patients with AMD; moreover, a phase IIIb/IV trial (ARIES) was also completed [63]. In VIEW 1 and VIEW 2 studies, authors found that all aflibercept groups were non-inferior to the monthly ranibizumab group by the end of the first year (Table 2) [61]. According to the results of the ARIES trial, the mean change in best-corrected visual acuity (BCVA) (ETDRS letters) from baseline to the end of the second year was 4.3 ± 13.4 in the early-start treat-and-extend (T&E) arm and 7.9 ± 11.9 in the late-start T&E arm (Table 2) [63]. To test monthly aflibercept intravitreal injections for DME, two phase III studies were performed over three years. When 2 mg of aflibercept was administered monthly, the mean BCVA gains from baseline to week 148 were 10.4 ± 14.2 in the VISTA and 10.3 ± 12.5 in the VIVID trial. However, aflibercept increased the risk of SOAEs compared with the control group. Serious cataracts, vitreous hemorrhage, and retinal detachment occurred in 3.2%, 2.5%, and 1% of patients in the monthly groups of the VISTA and VIVID studies, respectively (Table 2) [64]. In a study by Lee et al., the authors compared the risk of serious treatment-related adverse events (TAEs) between nAMD and DME patients receiving intravitreal aflibercept or ranibizumab. According to the results in patients with nAMD who received aflibercept, the risk of TAEs was lower than in patients receiving ranibizumab. Among DME patients, there was no difference in the risk of TAEs between the aflibercept and ranibizumab groups [65].

#### Brolucizumab

Brolucizumab (Beovu®; Novartis, Basel, Switzerland) is a novel FDA-approved anti-VEGF treatment for nAMD and DME, and it is the first single-chain antibody fragment that targets VEGF-A in these diseases (Fig. 1). An advantage of brolucizumab (26 kDa) is that it is much smaller than the FDA-approved aflibercept (115 kDa) or ranibizumab (48 kDa), which allows higher molar dosing less frequently [66].

Two phase III clinical studies, HARRIER and HAWK, have demonstrated that quarterly administered brolucizumab is non-inferior to the bimonthly administered aflibercept in efficacy to week 48, and this was maintained to week 96. Based on these promising results, similar effects can be achieved with fewer intravitreal injections of brolucizumab and with more frequent intravitreal injections of aflibercept [67]. In the MERLIN phase IIIa clinical trial, brolucizumab also showed non-inferiority to aflibercept in terms of BCVA change in nAMD. However, the incidences of retinal artery occlusion, retinal artery embolism, and retinal vein occlusion were higher in the brolucizumab group compared with the aflibercept group, leading to the early termination of the study (Table 1) [68]. These serious adverse events are most probably elicited by a strong immune reaction and thus limit the use of brolucizumab in everyday practice in spite of the marketing authorizations issued by the FDA and EMA. A phase III study (KINGFISHER) evaluated the efficacy and safety of monthly doses of brolucizumab in DME patients. The mean BCVA gain was 12.2 letters, and brolucizumab was non-inferior to aflibercept (Table 1) [69].

#### Faricimab

Faricimab (Vabysmo™; Roche, Basel, Switzerland) is an anti-angiopoietin-2/anti-VEGF bispecific antibody that inhibits two signaling pathways by binding and neutralizing both VEGF-A and angiopoietin-2 (Ang2) (Fig. 1). Ang2 is upregulated by hypoxia and VEGF, and its binding to the Tie2 receptor facilitates neovascularization by destabilizing the existing vasculature [70].

Two phase III clinical trials, TENAYA and LUCERNE, compared treatments with 6 mg of faricimab administered every 16 weeks and 2 mg of aflibercept administered every 8 weeks in treatment-naïve patients with nAMD. Mean gains in BCVA from baseline at week 48 were 5.8 and 6.6 letters in the TENAYA and LUCERNE studies in the faricimab arms, respectively (Table 2) [71]. Two phase III randomized, double-masked clinical trials were performed with bimonthly administered intravitreal faricimab injections for DME as well, where mean changes in BCVA from baseline at week 100 were 10.7 letters in the YOSEMITE trial and 10.9 letters in the RHINE trial (Table 2) [72].

#### Abicipar pegol

Abicipar pegol (Abicipar®; Allergan, Dublin, Ireland) is a novel anti-VEGF drug based on designed ankyrin repeat protein therapeutics (DARPin®; Molecular Partners, Zurich, Switzerland), which is a novel class of medications derived from natural ankyrin repeat proteins. Abicipar binds to its target with high affinity and specificity; thus, it is an antagonist of all isoforms of VEGF-A (Fig. 1).

In two phase III clinical trials (SEQUIA and CEDAR) [73], abicipar pegol was compared to ranibizumab in treatment-naïve patients with nAMD. Based on pooled data from 2-year results reported by Khurana et al., abicipar pegol showed similar efficacy to that of ranibizumab. At week 104, the proportions of patients with stable vision were 93.0% and 89.8% when abicipar pegol was administered every 8 and 12 weeks, respectively. Monthly dosed ranibizumab maintained stable vision in 94.4% of the patients. Based on the pooled data from the two studies, mean gains in BCVA were 7.8, 6.1, and 8.5 letters in the 8-weekly dosed 2 mg abicipar (2Q8), 12-weekly dosed 2 mg abicipar (2Q12), and 4-weekly dosed 0.5 mg ranibizumab (0.5Q4) groups, respectively. However, abicipar increased the rate of IOI more than ranibizumab. The overall incidence of IOI from baseline to week 104 was 16.2%, 17.6%, and 1.3% in the abicipar 2Q8, abicipar 2Q12, and ranibizumab 0.5Q4 arms, respectively (Table 1) [73]. In 2019, a smaller phase II study (MAPLE) was completed to evaluate the safety of intravitreal 2 mg abicipar for nAMD. In the MAPLE study, a lower rate of IOI was reported, with an overall incidence of 8.9%, which was attributed to the modified manufacturing process of abicipar pegol [74].

#### Conbercept

Conbercept (Lumitin®; Chengdu Kanghong Biotech Co., Ltd., Chengdu, China) is a recombinant fusion protein composed of the second Ig domain of VEGFR-1 and the third and fourth Ig domains of VEGFR-2 fused to the Fc region of human IgG1 and binds all isoforms of VEGF-A, VEGF-B, VEGF-C, and PlGF (Fig. 1). It has been approved in China since 2013 for the treatment of nAMD; however, it has not yet been approved in other countries.

Conbercept underwent a phase III, randomized clinical trial that compared intravitreal injections of 0.5 mg conbercept with intravitreal sham injections in nAMD. The mean gains in BCVA were 10.0 ± 14.0 letters during the first year in the 0.5 mg conbercept arm compared with the 8.8 ± 13.9 letters in the sham injection group (Table 2) [75]. In patients with DME, a phase III trial (SAILING) showed an improvement in BCVA of 8.2 ± 9.5 letters in patients who received laser photocoagulation followed by 0.5 mg conbercept intravitreal injections (Table 2) [76].

#### OPT-302

OPT-302/sozinibercept (Opthea, Victoria, Australia) is also a novel recombinant fusion protein consisting of the first three extracellular domains of VEGFR-3 fused to the Fc region of IgG1. It binds VEGF-C and -D, thus preventing their binding to the endogenous VEGFR-2 and VEGFR-3 (Fig. 1).

In a phase IIb clinical trial, OPT-302 was combined with ranibizumab to study its efficacy in nAMD patients, and significantly better vision gain was observed with 2 mg of OPT-302 combined with ranibizumab than in the sham group receiving sham intravitreal injections plus intravitreal ranibizumab (Table 2) [77]. Another phase II trial was conducted to evaluate the efficacy of 2 mg OPT-302 combined with 2 mg aflibercept in DME patients. According to the results, the mean change in BCVA from baseline was 5.9 and 6.1 letters in the OPT-302 and sham groups, respectively (Table 2) [78]. To date, two phase III clinical trials are in progress with OPT-302.

Currently, anti-VEGF therapy is the first-line treatment for conditions with ocular neovascularization because several anti-VEGF therapeutics show good efficacy based on VA and BCVA results from various studies conducted during the past two decades. Compared to laser photocoagulation and PDT, therapies targeting VEGF perform better, and considering the AEs occurring during treatment, they are safer to use.

Despite the widespread success of anti-VEGF therapy, it can cause several SOAEs following the intravitreal administration, including endophthalmitis, cataract, retinal detachment, and elevation in intraocular pressure. Some studies also suggest that the use of anti-VEGF agents is associated with the development of glaucoma [79, 80].

**Fig. 3:**
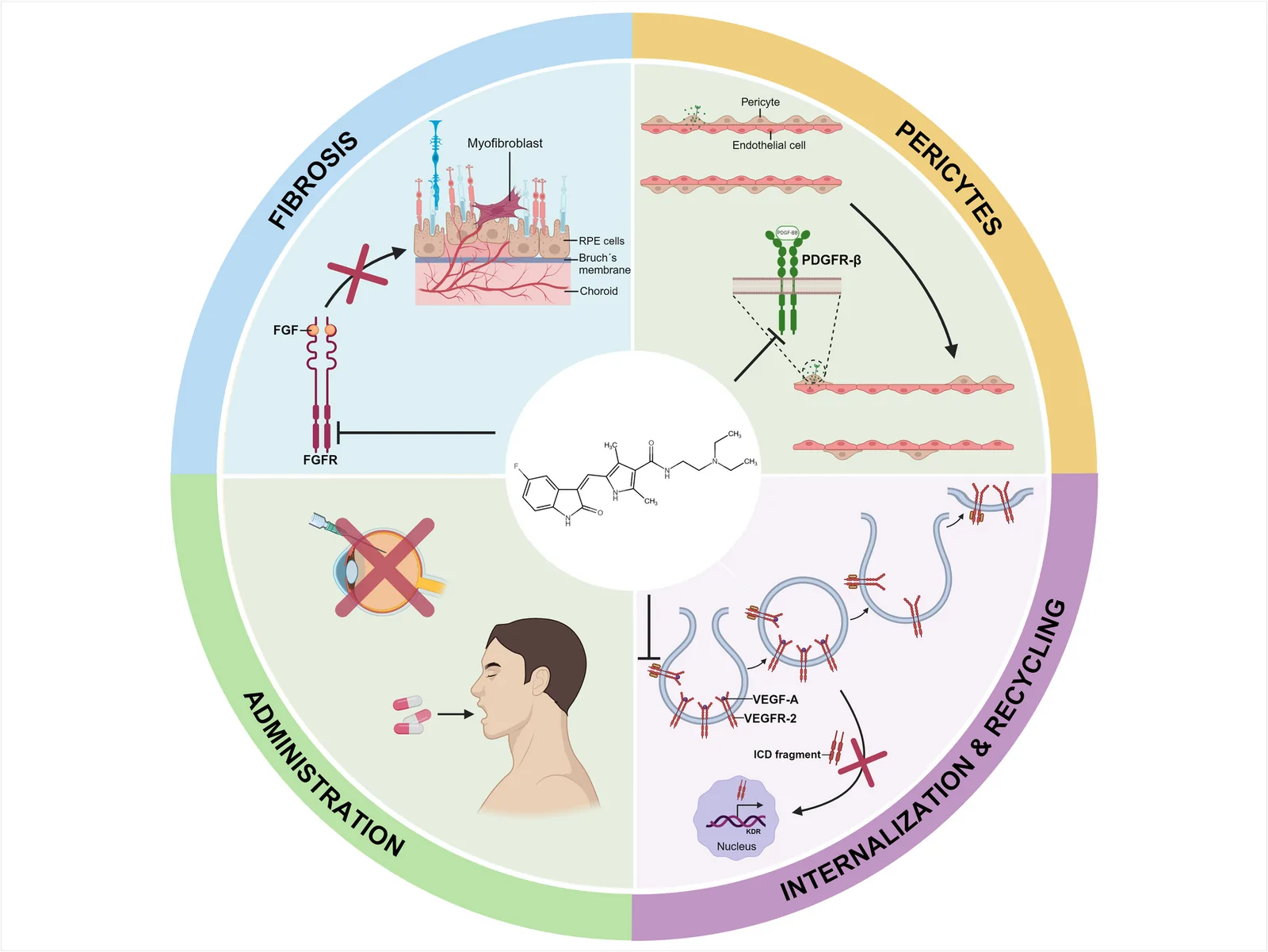
Schematic diagram of the benefits provided by sunitinib, a small-molecule TKI over anti-VEGF therapy. FGF, fibroblast growth factor; FGFR, fibroblast growth factor receptor; ICD, intracellular domain; PDGF-BB, platelet-derived growth factor BB; PDGFR-β, platelet-derived growth factor receptor β; RPE, retinal pigment epithelium; VEGF-A, vascular endothelial growth factor A; VEGFR-2, vascular endothelial growth factor receptor 2. Created in BioRender.com

Moreover, unfortunately, approximately 20% of nAMD patients [81, 82] and approximately 15% of PDR patients are non-responders to anti-VEGF agents [83]. One of the reasons for anti-VEGF resistance in endothelial cells is that anti-VEGF treatment may lead to the upregulation of the platelet-derived growth factor (PDGF)-BB. PDGF-BB secreted by endothelial cells binds to PDGF receptor β (PDGFR-β), which is expressed on pericytes, vascular endothelial cells, and RPE. PDGF-BB is a strong chemoattractant for pericytes. Thus, through PDGF-BB/PDGFR-β signaling, endothelial cells recruit pericytes expressing the receptor (Fig. 3) [84, 85]. These cells promote the proliferation of endothelial cells and enable cell survival independently of VEGF [86]. In addition, PDGF-BB/PDGFR-β signaling enhances VEGF secretion by pericytes; thus, anti-VEGF therapy becomes less efficient [87]. Furthermore, some studies suggest that the endothelial neuropilin-1 (NRP-1), a co-receptor for VEGF, PlGF, and PDGF, regulates the vascular permeability and vascular remodeling mechanisms during angiogenesis [88, 89]. NRP-1 may work VEGF-independently, which could play a role in anti-VEGF resistance. Another study identified semaphorin 3 A (SEMA3A) as a potential biomarker for DR, since the SEMA3/NRP-1 signaling has also been associated with increased vascular permeability [90]. These findings suggest that targeting the abovementioned biomarkers alongside VEGF could increase the efficacy of anti-VEGF therapy and decrease the rate of non-responders. A further phenomenon should also be mentioned, which is the response loss, also known as tachyphylaxis, that can decrease or gradually eliminate the desired effect of anti-VEGF agents. In this case, patients who had a good initial response became resistant to further treatment [91].

### Gene therapy

Gene therapy is a continuously developing medical treatment that can offer visual improvements for individuals with various ocular diseases. During gene therapy, genetic material is introduced, removed, or modified inside the cells of the patients. Thus, diseases can be treated or even prevented. In recent years, several gene therapy products have been in clinical development to treat conditions linked to ocular neovascularization, specifically nAMD and PDR. The goal of gene therapy products for nAMD and PDR is to introduce the genetic code of anti-VEGF agents into the cells of the eye with a single injection. This way, the repeated intravitreal injections could be avoided since the genetic code would be inserted permanently and would produce therapeutic proteins at a constant rate.

The strategy for delivering genetic material is often based on viral vectors. Lentiviruses and retroviruses are single-stranded RNA viruses that can integrate their genetic code into the host genome. This way, long-term transgene expression can be achieved in both dividing and non-dividing cells. However, several potential adverse effects have to be considered, including gene silencing, overexpression of the transgene, and, most importantly, insertional mutagenesis, which can cause tumor formation [92, 93]. Adenoviruses are double-stranded DNA viruses that can infect dividing and non-dividing cells. The risk of insertional mutagenesis is much lower since their genetic material cannot be inserted into the host genome; instead, it remains in the episomes. However, the disadvantage of adenoviral vectors is that the genetic material in episomes is diluted over time in dividing cells. Moreover, adenoviral vectors can activate a strong immune response, resulting in the removal of cells expressing adenoviral proteins. Therefore, adenoviral vectors are not commonly used for retinal gene therapy [94]. Instead, adeno-associated virus (AAV) vectors constitute the method of choice. These vectors can infect both dividing and non-dividing cells, and their genetic material remains mainly in the episomes. They require a helper virus for replication; thus, AAVs can elude the host immune response and are non-pathogenic [95].

With gene therapy, several intravitreal administrations of anti-VEGF agents per year can be avoided or reduced; thus, the risk of OAEs would be much lower since most of the gene therapy products are designed as a single injection per year. Furthermore, successful gene therapy offers a long-term positive effect in patients, as evidenced by the clinical trials: some of the gene therapy products resulted in sustained levels of intraocular anti-VEGF proteins [96]. These could significantly improve patient compliance. However, most of the gene therapy products are designed as treatments to complement anti-VEGF therapy. Thus, it may not be possible to phase out protein-based anti-VEGF therapy completely. While gene therapy products offer promising alternatives for the treatment of nAMD and PDR, it is crucial to acknowledge that they are relatively new approaches and that there is limited evidence base regarding long-term risk and adverse events. This persisting burden points to a substantial advantage over all other therapeutic approaches that small-molecule TKIs can boast, provided that their untoward effects can be reduced [97, 98].

### Recent clinical trials investigating gene therapies

#### Ixo-vec (ADVM-022)

Ixo-vec, formerly referred to as ADVM-022 (Adverum Biotechnologies; Redwood City, CA, USA), is an intravitreal therapy for the treatment of nAMD. It uses an engineered AAV capsid (AAV2.7m8) that contains the genetic material of aflibercept. As an anti-VEGF agent, it is capable of inhibiting not only VEGF-A but also VEGF-B and PlGF.

In the OPTIC phase I study, a single intravitreal injection of Ixo-vec was administered to patients who had previously received anti-VEGF treatment. Gene therapy resulted in sustained levels of intraocular aflibercept, maintenance of VA, and a significant reduction in anti-VEGF rescue injections. However, several SOAEs have been reported, including cataracts, retinal detachment, and uveitis (Table 3) [96]. The company announced that in the currently active phase II LUNA trial, both doses of Ixo-vec maintained visual endpoints at week 52, and there were no treatment-related SOAEs (Table 3) [99].

**Table 3:** Summary of key clinical trials on visual outcomes and occurring adverse events following gene therapy for age-related macular degeneration and diabetic retinopathy

Therapeutic modality	Approved indicatio	Study ref	Study design	No. of patients	Group	BCVA (ETDRS letters)		OAEs	Trial registration number on clinicaltrials	Status
						Baseline	Change			
Ixo-vec	None	OPTIC [96]	Phase I	Total: 30	2 × 10^11^ viral genome/eye	64.7 (7.6)	0.2 (N.A.)	Cataract (7%)^a,b^, dry AMD (7%)^a,b^	NCT03748784	Complete
					6 × 10^11^ viral genome/eye	65.0 (7.8)	− 0.2 (N.A.)	Cataract (7%)^a,b^, retinal detachment (7%)^a,b^, uveitis (7%)^a,b^		
		LUNA [99]	Phase II	Total: 60	6 × 10^10^ viral genome/eye	72.9 (8.8)	−2.1 (N.A.)^a^ LS mean	N.A	NCT05536973	Active
					2 × 10^11^ viral genome/eye	71.8 (6.4)	−1.8 (N.A.)^a^ LS mean			
RGX-314	None	NCT03066258 [100]	Phase I/II	Total: 42	3 × 10^9^ genome copies/eye	55.7 (N.A.)	− 7.6 (N.A.)	Conjunctival hemorrhage (33%), inflammation (67%), retinal hemorrhage (67%), VA reduced (50%), intraocular pressure increased (17%), eye pain (17%), photopsia (33%), vision blurred (17%)	NCT03066258	Completed
					1 × 10^10^ genome copies/eye		1.2 (N.A.)	Conjunctival hemorrhage (50%), retinal pigmentary changes (50%), inflammation (50%), VA reduced (50%), intraocular pressure increased (17%), eye pain (17%), photopsia (17%), vision blurred (17%), conjunctival hyperemia (17%)		
					6 × 10^10^ genome copies/eye		14.0 (N.A.)	Conjunctival hemorrhage (67%), retinal pigmentary changes (50%), inflammation (50%), retinal hemorrhage (17%), VA reduced (17%), intraocular pressure increased (33%), retinal degeneration (17%), conjunctival hyperemia (33%)		
					1.6 × 10^11^ genome copies/eye		0.9 (N.A.)	Conjunctival hemorrhage (75%), retinal pigmentary changes (100%), inflammation (25%), retinal hemorrhage (25%), VA reduced (8%), eye irritation (25%), eye pain (25%), retinal degeneration (17%), photopsia (8%)		
					2.5 × 10^11^ genome copies/eye		− 3.8 (N.A.)	Conjunctival hemorrhage (93%), retinal pigmentary changes (93%), inflammation (17%), retinal hemorrhage (25%), VA reduced (8%), intraocular pressure increased (33%), eye irritation (33%), eye pain (17%), retinal degeneration (33%), photopsia (17%), vision blurred (25%), conjunctival hyperemia (17%)		
		AAVIATE [102]	Phase II	Total: 116	2.5 × 10^11^ genome copies/eye	75.1	1.3 (N.A.)	N.A	NCT04514653	Active
				Control: 10	5 × 10^11^ genome copies/eye	71.9	1.7 (N.A.)	N.A		
					1 × 10^12^ genome copies/eye	72.8	1.0 (N.A.)	N.A		
					Ranibizumab	72.7	N.A	N.A		
		ALTITUDE [101]	Phase II	Total: 60	2.5 × 10^11^ genome copies/eye	78.1 (N.A.)	N.A	Conjunctival hyperemia (26.7%), conjunctival hemorrhage (20%)	NCT04567550	Active
				Control: 10	5 × 10^11^ genome copies/eye (NAb-) AAV8 neutralizing antibody NEGATIVE	82.1 (N.A.)	N.A	Conjunctival hyperemia (33.3%), conjunctival hemorrhage (13.3%), intraocular inflammation (20%)		
					5 × 10^11^ genome copies/eye (Nab +)	81.3 (N.A.)	N.A	Conjunctival hyperemia (20%), conjunctival hemorrhage (5%)		
					Observational control	84.5 (N.A.)	N.A	N.A		
4D-150	None	PRISM [105]	Phase I/II	Total: 45	1 × 10^10^ vector genomes/eye	73.0 (8.8)	N.A	N.A	NCT05197270	Active
					3 × 10^10^ vector genomes/eye	71.0 (9.9)	6.0 (N.A.)			
		SPECTRA [106]	Phase II	Total: 22	5 × 10^9^ vector genomes/eye	68.0 (N.A.)	N.A	N.A	NCT05930561	Active
					1 × 10^10^ vector genomes/eye	62.0 (N.A.)	7.1 (N.A.)			
					3 × 10^10^ vector genomes/eye	63.0 (N.A.)	8.4 (N.A.)			
AAV2-sFLT01	None	NCT01494805 [107]		Total: 8	1 × 10^10^ vector genome/eye	35.7 (N.A.)	3.7 (N.A.)	Cataract (33.3%) retinal hemorrhage (33.3%)	NCT01494805	Completed
				Control: 2	1 × 10^11^ vector genome/eye	48.0 (N.A.)	−9 (N.A.)			
					Non-gene therapy	33.5 (N.A.)	0 (N.A.)	Retinal hemorrhage (50%)		
		NCT01024998 [108]	Phase I	Total: 19	2 × 10^8^ vector genomes	25.2 (N.A.)	N.A	Cataract (33%)	NCT01024998	Completed
					2 × 10^9^ vector genomes			Eye discharge (33%), retinal hemorrhage (67%), subretinal fibrosis (33%), subretinal fluid (33%), vitreous detachment (33%)		
					6 × 10^9^ vector genomes		N.A	Conjunctival hyperemia (100%), eye pain (33%), eyelids pruritus (33%), vitreous floaters (33%)		
					2 × 10^10^ vector genomes		N.A	cataract (10%), conjunctival hemorrhage (60%), conjunctival hyperemia edema (20%), corneal deposits (10%), eye discharge (10%), iritis (10%), retinal hemorrhage (10%), retinal tear (10%)a, subretinal fibrosis (10%), visual impairment (10%), vitreous floaters (10%), vitritis (10%)		
HMR59 (AAVCAGsCD59)	None	NCT03144999 [109]	Phase I	Total: 19	Low dose (3.56 × 10^10^ viral genome/eye)	N.A	N.A		NCT03144999	Completed
					Intermediate dose (1.07 × 10^11^ viral genome/eye)	N.A	N.A	Ocular inflammation (33.3%), anterior chamber inflammation (33.3%), intraocular pressure increased (33.3%), optic nerve disorder (33.3%)		
					High dose (3.56 × 10^11^ viral genome/eye)	N.A	N.A	Ocular inflammation (36.4%), vitritis (36.4%), VA reduced (27.3%), intraocular pressure increased (9.1%), optic nerve disorder (9.1%)		
		NCT03585556 [110]	Phase I	Total: 25	Low dose (3.56 × 10^11^ viral genome/eye)	67.5 (N.A.)	N.A	Ocular inflammation (13.6%), uveitis (9.1%), anterior chamber cell (4.5%), VA reduced (9.1%), intraocular pressure increased (4.5%), keratic precipitates (4.5%), vitreal cells (4.5%), subretinal fibrosis (9.1%), optic disc hemorrhage (9.1%)	NCT03585556	Completed
					High dose (1.071 × 10^12^ viral genome/eye)	68.0 (N.A.)	N.A	Ocular inflammation (33.3%), eye inflammation (33.3%)		
		NCT04358471 [111]	Phase II	N.A	3.56 × 10^11^ viral genome/eye	N.A	N.A	N.A	NCT04358471	Withdrawn
					1.071 × 10^12^ viral genome/eye					
					Sham					

Data are expressed as mean (standard deviation). *BCVA*, best-corrected visual acuity; *ETDRS*, Early Treatment of Diabetic Retinopathy Study; *OAEs*, ocular adverse events; *N.A.*, not available. ^a^Serious OAEs. ^b^Treatment-related adverse events

#### RGX-314

RGX-314 (REGENXBIO; Rockville, MD, USA) is currently under clinical development as a one-time treatment for nAMD and DR. AAV serotype 8 (AAV8) serves as a vector containing the transgene of a monoclonal antibody fragment, similar to ranibizumab, which is capable of neutralizing VEGF.

In patients who underwent a phase I/II clinical trial, the mean baseline BCVA was maintained or improved in the case of all doses except the lowest, and there was a striking reduction in the number of anti-VEGF injections at the higher doses (Table 3) [100]. In the currently ongoing phase II trial (ALTITUDE), the efficacy of RGX-314 is being evaluated in patients with DR. According to 1-year data announced by the company, NPDR patients demonstrated clinically meaningful improvements in disease severity, and there were no drug-related SOAEs (Table 3) [101]. The phase II AAVIATE trial is currently evaluating RGX-314 by suprachoroidal delivery in nAMD patients. According to interim data, RGX-314 therapy resulted in stable vision, and it reduced anti-VEGF injection by 80%. No treatment-related serious AEs were observed [102]. Currently, in the ongoing phase II/III ATMOSPHERE and phase III ASCENT trial, the subretinal delivery of RGX-314 is being evaluated [103, 104].

#### 4D-150

This gene therapy is under clinical development by 4D Molecular Therapeutics (Emeryville, CA, USA) as a single-dose intravitreal injection for the treatment of DME. In 4D-150, the AAV vector R100 contains a transgene that expresses an aflibercept-like protein targeting VEGF-A, VEGF-B, and PlGF, and a VEGF-C targeting miRNA; thus, this gene therapy product is capable of neutralizing multiple growth factors at the same time.

In the ongoing phase I/II PRISM study, 4D-150 is being evaluated in nAMD patients in two different doses, and according to interim results, the mean change in BCVA from baseline was 6.0 letters at week 48 in the high-dose (3 × 10^10^ vector genomes/eye) group. Furthermore, to date, there were no treatment-related serious AEs reported [105]. According to interim data from the other ongoing phase II SPECTRA study evaluating 4D-150 in DME patients, the mean gain of BCVA from baseline was 8.4 letters in the high-dose group, and no intraocular inflammation was observed at any dose level [106].

#### AAV2-sFLT01

This gene therapy product, developed by Sanofi/Genzyme (Cambridge, MA, USA) to treat nAMD, uses an AAV2 as a vector containing the genetic material of a VEGF trap protein sFLT01 that can neutralize VEGF.

Two phase I clinical studies have been completed studying AVV2-sFLT01 administered as a single intravitreal injection. In a dose escalation trial assessing the safety of the gene therapy product in patients with nAMD, two out of three patients dropped out after month 18 in the high-dose group for reasons unrelated to the study, and there were no SOAEs between weeks 12 and 52 in any of the cohorts (Table 3) [107]. In the second phase I dose-escalating study, the only SOAE through 6 years was retinal tear in the high dose group (2 × 10^10^ vector genomes) (Table 3) [108]. To date, there have been no ongoing phase II trials with AAV2-sFLT01.

#### HMR59

HMR59 (AAVCAGsCD59) was developed by Hemera Biosciences (Waltham, MA, USA), and later, Janssen Pharmaceuticals (Beerse, Belgium) acquired the rights to the gene therapy product HMR59. Geographic atrophy is a complication of advanced AMD that affects millions of people worldwide. In geographic atrophy, cells in the macula are destroyed due to the overactivity of the complement. HMR59 must be administered intravitreally, and the transgene product CD59 blocks the membrane attack complex (MAC), the final step of the complement cascade. Thus, HRM59 can help prevent further retinal damage and the development of nAMD.

To date, there have been two completed phase I studies with this gene therapy product, and no SOAEs related to HMR59 have occurred during the follow-up months (Table 3) [109, 110]. Despite the good safety outcomes, a planned phase II study with HMR59 was later withdrawn, and to date, there have been no other ongoing trials [111].

### Small-molecule tyrosine kinase inhibitors

Besides the inhibition of VEGF by biotechnological agents or gene therapy products, another method to counterbalance the effect of upregulated VEGF signaling in the eye is to inhibit the activity of VEGFRs and other receptor tyrosine kinases by small-molecule TKIs. This type of approach may offer several advantages over anti-VEGF therapy (Fig. 3).

Many TKIs are pan-VEGFR inhibitors, as they can inhibit all isoforms of VEGFRs, whereas many anti-VEGF agents can block only VEGF-A/VEGFR-2 signaling. Moreover, several TKIs target not only one but a variety of growth factor receptor families, for example, VEGFRs, PDGFRs, fetal liver tyrosine kinase 3 (Flt-3), and fibroblast growth factor receptors (FGFR). These “multireceptor” TKIs, such as sunitinib, vorolanib, nintedanib, and axitinib, have a huge benefit over anti-VEGF therapy in ophthalmological indications since they are combined anti-VEGFR-2/PDGFR-β inhibitors inhibiting the tyrosine kinase activity of both receptors at the same time, thus decreasing angiogenesis more effectively (Fig. 1) [112–114]. In this way, these TKIs can be efficient in patients with nAMD and PDR who were non-responders to anti-VEGF therapy.

Besides their role in retinal fibrosis, FGFR-1 and −2 expressed on endothelial cells contribute to pathological angiogenesis in the eye as well (Fig. 3) [115]. Activation of FGFR signaling pathways is responsible for the survival, proliferation, and differentiation of endothelial cells. Moreover, it induces the recruitment of pericytes, vascular smooth muscle cells, and monocytes, thus affecting vascular integrity [116]. According to a study in mice, fibroblast growth factor-2 (FGF-2) promoted CNV, and it is the main pro-angiogenic ligand required for endothelial FGFR signaling [117]. Thus, inhibiting FGFRs may also be beneficial in the treatment of pathological angiogenesis in ophthalmological indications.

Furthermore, the binding of VEGF to VEGFR-2 induces cleavage within the receptor, and the generated intracellular domain (ICD) fragment translocates to the nucleus. The ICD fragment, in turn, enhances the transcription of VEGFR-2 (Fig. 3) [118, 119]. The other fraction of the internalized receptors is recycled back into the plasma membrane [120]. Recycled receptors can be activated again by ligands on the cell surface. In contrast, those receptors which were inhibited by TKIs before internalization cannot be activated after recycling because TKIs may remain bound to recycled VEGFR-2 (Fig. 3). Moreover, some TKIs can largely prevent the cleavage of the C-terminal portion of VEGFR-2 (such cleavage being activation-dependent); thus, upregulation of VEGFR-2 expression can be avoided [121].

Some TKIs currently undergoing preclinical and clinical trials can be administered orally to nAMD and DR patients instead of intravitreal injections, which is also a major advantage (Fig. 3) [97, 122–124]. This potential route of administration would eliminate all OAEs (e.g., endophthalmitis, hemorrhages, cataracts, retinal detachment) related to intravitreal anti-VEGF therapy and would drastically increase patient compliance.

TKIs have the potential to provide further benefits. The production costs of a recombinant monoclonal antibody, which requires living cells, are much higher than those of a small-molecule TKI that can be synthesized via more simple chemical reactions. Furthermore, generic TKIs against nAMD and PDR will be easier to introduce into the market from a regulatory point of view and will be much less expensive to produce, in line with the general consideration that the development and manufacturing of generics remarkably cost less than that of biosimilar products [125].

### Recent clinical trials investigating small-molecule tyrosine kinase inhibitors

#### Sunitinib

Sunitinib is an oral small-molecule multireceptor tyrosine kinase inhibitor that inhibits VEGFR-1, −2, −3, PDGFR-α, -β, Flt-3, and stem cell factor receptor (c-Kit), and it is a moderate inhibitor of FGFRs (Fig. 1) [126]. Its currently approved indications include several cancer types, such as metastatic renal cell carcinoma, well-differentiated pancreatic neuroendocrine cancers, and gastrointestinal stromal tumors [127, 128]. Although serious systemic adverse events (SSAEs) of sunitinib related to its oral dosing are tolerable in patients suffering from otherwise incurable cancer, these AEs exclude the oral administration in ophthalmological indications.

In clinical trials testing oral sunitinib in patients with cancer, gastrointestinal AEs, including diarrhea, nausea, hypertension, alanine transaminase, and aspartate transaminase elevations, were quite common [129]. These AEs also appear in clinical studies testing oral sunitinib for ophthalmological conditions. Hepatotoxicity is a SSAE that occurs during the oral administration of oral sunitinib. According to Paech et al., sunitinib is hepatotoxic in a concentration-dependent manner, and this effect is associated with mitochondrial dysfunction and inhibition of glycolysis at concentrations that can occur in the liver of some patients [130].

#### GB-102

To avoid SSAEs occurring after the oral administration of sunitinib, GB-102 (GrayBug Vision; Redwood City, CA, USA) was developed. This investigational product contains sunitinib-malate and is an intravitreal injection of a novel depot formulation patented by GrayBug Vision, which is intended to be used twice per year for the treatment of nAMD. The molecule is encapsulated within bioerodible polymer nanoparticles that form an implant-like depot in the inferior vitreous and slowly degrade over time; thus, the release of the drug becomes much slower than that of orally administered sunitinib.

ADAGIO, a phase I/IIa clinical study, was conducted in 2019 to test GB-102 in patients with nAMD. According to the results announced by GrayBug Vision, the study met its primary endpoint; there were no drug-related SOAEs, and the drug was well tolerated with no dose-limiting toxicities. In 88% and 68% of the patients, there was a long-term positive pharmacodynamic effect at 3 and 6 months, respectively (Table 4) [131]. The company compared 1 mg and 2 mg doses of GB-102 in a phase IIa uncontrolled trial in DME patients as well. Based on these results, the mean changes in BCVA were − 10.4 letters and − 16.7 letters in the 1 mg and 2 mg arms, respectively, at 6 months. Serious and non-serious OAEs were also detected during the trial, and the rates of AEs were higher in the 2 mg group compared with the 1 mg group (Table 4) [132]. In 2021, GB-102 was tested in a phase IIb, randomized, active-controlled trial (ALTISSIMO) to evaluate the safety and duration of repeated intravitreal injections of 1 mg and 2 mg of GB-102 injected every 6 months compared with 2 mg of aflibercept injected every 2 months in nAMD patients. Patients in the 2 mg GB-102 arm were switched to 1 mg for their second dose after the results of an interim safety analysis. 48% of patients did not require supportive therapy for at least 6 months, and 62% of patients for at least 4 months. The 1 mg dose performed better than the 2 mg dose. However, the mean changes in BCVA were − 7.4 ± 4.7 and 1.8 ± 1.0 letters in the 1 mg of GB-102 and 2 mg of aflibercept arm through 12 months, respectively. The majority of drug-related AEs were mild to moderate, and no serious AEs were detected in the 1 mg GB-102 arm. However, the OAEs rates were higher in both the 1 mg and 2 mg GB-102 arms than in the aflibercept arm (Table 4) [133, 134]. In these recent studies with GB-102, there were no drug-related SSAEs even though it is a small-molecule TKI; however, OAEs were quite common, including vitreous floaters, retinal hemorrhages, cataracts, and VA reduction.

**Table 4:** Summary of key clinical trials on visual outcomes and occurring serious adverse events following treatment with small-molecule tyrosine kinase inhibitors for age-related macular degeneration and diabetic retinopathy

Therapeutic modality	Approved indications	Study (Ref.)	Study design	No. of patients	Group	BCVA (ETDRS letters)		SOAEs^a^	SSAEs^b^	Trial registration number on clinicaltrials.gov	Status
						Baseline	Change				
GB-102	None	ADAGIO [131]	Phase I/IIa	Total: 32	GB-102 0.25 mg	N.A	N.A			NCT03249740	Completed
					GB-102 0.5 mg						
					GB-102 1.0 mg						
					GB-102 2.0 mg						
		Macular Edema Study [132]	Phase lla	Total: 21	GB-102 1.0 mg	N.A	− 10.4 (N.A.)		Acute myocardial infarction (10%)	NCT04085341	Completed
					GB-102 2.0 mg	N.A	− 16.7 (N.A.)	Corneal edema (9.1%), visual impairment (9.1%)	Gastrointestinal hemorrhage (9.1%)		
		ALTISSIMO [133]	Phase IIb	Total: 50	GB-102 1.0 mg	70.8 (11.5)	− 7.4 (4.7)		Pneumonia (4.8%), sepsis (4.8%)	NCT03953079	Completed
				Control: 12	GB-102 2.0 mg	70.7 (9.9)	− 5.1 (4.4)	Retinal detachment (4.6%)	atrial fibrillation (4.6%), urosepsis (4.6%), acute myeloid leukemia (4.6%), bladder cancer (4.6%), metastatic gastric cancer (4.6%)		
					Aflibercept 2.0 mg	69.6 (14.7)	1.8 (1.0)		intraductal proliferative breast lesion (7.8%)		
Oral vorolanib	None	NCT01674569 [97]	Phase I	Total: 35	Vorolanib 50.0 mg alternated	62.4 (12.6)	3.8 (9.6)		Chest pain (8.6%), congestive heart failure (2.9%), multiple myeloma (2.9%), acute renal failure (2.9%), abdominal discomfort 2.9%)^c^, abdominal pain (2.9%)^c^, diarrhea (11.4%)^c^, nausea (8.6%)^c^, fatigue (11.5%)^c^, elevated alanine aminotransferase (ALT) (8.6%)^c^, elevated aspartate aminotransferase (AST) (17.1%)^c^, liver function test abnormal (2.9%)^c^, weight decreased (2.9%)^c^, decreased appetite (2.9%)^c^, muscle spasms (5.7%)^c^, dysgeusia (5.7%)^c^, dizziness (2.9%)^c^, pollakiuria (2.9%)^c^, accelerated hypertension (2.9%)^c^	NCT01674569	Completed
					Vorolanib 50.0 mg daily						
					Vorolanib 100.0 mg alternated						
					Vorolanib 100.0 mg daily						
					Vorolanib 200.0 mg daily						
					Vorolanib 300.0 mg daily						
		APEX [98]	Phase II	Total: 157	Vorolanib 50.0 mg	72.3 (9.4)	0.2 (4.1)		Myocardial infarction (2.5%), sepsis (2.5%), urinary tract infection (2.5%), hip fracture (2.5%), bladder cancer (2.5%), basal cell carcinoma (2.5%), endometrial cancer (2.5%), esophageal carcinoma (2.5%), squamous cell carcinoma (2.5%), diarrhea (2.5%)^c^, vomiting (2.5%)^c^, elevated ALT and AST (20%)^c^	NCT02348359	Terminated
				Control: 39	Vorolanib 100.0 mg	71.2 (12.2)	− 0.9 (6.6)	Endophthalmitis (2.6%)	Sudden cardiac death (2.6%), influenza (2.6%), dehydration (2.6%), hypokalemia (2.6%), bladder cancer (2.6%), angioedema (2.6%), deep vein thrombosis (2.6%), fatigue (2.6%)^c^, elevated ALT, and AST (28.2%)^c^		
					Vorolanib 200.0 mg	72 (13.2)	1.7 (5.6)		Lower gastrointestinal hemorrhage (2.6%), chest pain (2.6%), B cell lymphoma (2.6%), hypertensive crisis (2.6%), diarrhea (5.1%)^c^, vomiting (2.6%)^c^, fatigue (2.6%)^c^, elevated ALT and AST (23.1%)^c^		
					Placebo	68.8 (12.4)	− 0.3 (10.6)		pneumonia (2.6%), syncope (2.6%), elevated ALT, and AST (5.1%)^c^		
		NCT02452385 [136]	Phase I	Total: 41	Vorolanib 25.0 mg	43.6 (18.0)	4.3 (N.A.)		Elevated ALT (18.8%)^c^, elevated AST (18.8%)^c^, elevated blood pressure (25%)^c^, leukopenia (6.3%)^c^, QT interval prolongation (12.5%)^c^, thrombocytopenia (12.5%)^c^, neutropenia (6.3%)^c^	NCT02452385	Suspended
					Vorolanib 50.0 mg	54.1 (14.9)	8.5 (N.A.)		Elevated ALT (56.3%)^c^, elevated AST (50%)^c^, elevated blood pressure (37.5%)^c^, leukopenia (18.8%)^c^, QT interval prolongation (25%)^c^, thrombocytopenia (6.3%)^c^, neutropenia (18.8%)^c^, hair color changes (25%)^c^		
					Vorolanib 75.0 mg	45.6 (16.9)	12.0 (N.A.)		Elevated ALT (62.5%)^c^, elevated AST (50%)^c^, elevated blood pressure (37.5%)^c^, leukopenia (37.5%)^c^, QT interval prolongation (12.5%)^c^, thrombocytopenia (37.5%)^c^, neutropenia (25%)^c^, hair color changes (25%)^c^		
					Vorolanib 100.0 mg	33.0 (N.A.)	N.A				
		NCT03710863 [137]	Phase II	Total: 64	Vorolanib 25.0 mg	N.A	N.A	N.A	N.A	NCT03710863	Unknown Status
					Vorolanib 50.0 mg						
EYP-1901	None	DAVIO1 [171]	Phase I	Total: 17	EYP-1901 0.44 mg	69 (N.A.)	− 5.4 (N.A.)			NCT04747197	Completed
					EYP-1901 1.03 mg						
					EYP-1901 2.06 mg						
					EYP-1901 3.09 mg						
		DAVIO2 [138]	Phase II	Total: 161	EYP-1901 2.0 mg	73.9 (N.A.)	1.0 (N.A.)	N.A	N.A	NCT05381948	Completed
				Control: 54	EYP-1901 3.0 mg	74.9 (N.A.)	0.9 (N.A.)				
					Aflibercept 2.0 mg	73.4 (N.A.)	1.3 (N.A.)				
		VERONA [139]	Phase II	Total: 25	EYP-1901 1.3 mg	N.A	N.A	N.A	N.A	NCT06099184	Active
				Control: N.A	EYP-1901 2.7 mg	N.A	7.1 (N.A.)				
					Aflibercept 2.0 mg	N.A	3.2 (N.A.)				
		PAVIA [140]	Phase II	Total: 77	EYP-1901 2.1 mg	N.A	N.A	N.A	N.A	NCT05383209	Completed
				Control: N.A	EYP-1901 3.1 mg						
					Sham						
CLS-AX	None	OASIS [143]	Phase I/IIa	Total: 27	CLS-AX 0.03 mg	59.0 (16.4)	2.2 (4.9)			NCT04626128	Completed
					CLS-AX 0.1 mg	65.6 (8.7)	− 6.3 (9.2)				
					CLS-AX 0.5 mg	58.5 (13.7)	− 2.1 (4.5)				
					CLS-AX 1.0 mg	65.8 (8.3)	0.3 (3.3)				
		ODYSSEY [144]	Phase IIb	Total: N.A	CLS-AX 1.0 mg	N.A	N.A	N.A	N.A	NCT05891548	Completed
				Control: N.A	Aflibercept 2.0 mg						
OTX-TKI	None	NCT04989699 [145]	Phase I	Total: 21	OTX-TKI 0.6 mg + aflibercept 2.0 mg	N.A	− 1.0 (N.A.)	N.A	N.A	NCT04989699	Completed
				Control: N.A	Aflibercept 2.0 mg	N.A	2.0 (N.A.)				
		NCT06223958 [146]	Phase III	Total: N.A	OTX-TKI N.A	N.A	N.A	N.A	N.A	NCT06223958	Active
				Control: N.A	Aflibercept 2.0 mg						

Data are expressed as mean (standard deviation). *BCVA*, best-corrected visual acuity; *ETDRS*, Early Treatment of Diabetic Retinopathy Study; *SOAEs*, serious ocular adverse events; *SSAEs*, serious systemic adverse events; *N.A.*, not available. ^a^SOAEs occurring in ≥ 1% of patients in study eye. ^b^SSAEs occurring in ≥ 2% of patients. ^c^Treatment-related adverse events

#### Vorolanib

Vorolanib (X-82, CM082) is an oral small-molecule tyrosine kinase inhibitor, structurally similar to sunitinib. It inhibits all the isoforms of VEGFR and PDGFR (Fig. 1) [98]. It has been designed to have a shorter half-life and limited tissue accumulation compared with sunitinib to minimize systemic AEs in cancer patients.

According to the first phase I clinical trial, orally administered vorolanib in patients with advanced solid tumors has met these expectations [135]. In the phase I uncontrolled dose-escalation study in 25 nAMD participants who completed the 24 weeks of treatment, the mean BCVA change from baseline was 3.8 ± 9.6 letters. Fifteen of the 25 participants did not require rescue ranibizumab injections. However, 10 of 35 patients (29%) did not complete the 24-week endpoint, of whom 6 (17%) were withdrawn due to AEs potentially related to vorolanib. The most common AEs were diarrhea (17.1%), nausea (14.3%), fatigue (14.3%), and transaminase elevation (11.4%) (Table 4) [97]. Given its promising efficacy in phase I trials, a further placebo-controlled phase II trial (APEX) was conducted in patients with nAMD, who had received at least two intravitreal injections of anti-VEGF prior to the study enrolment. In the 50 mg, 100 mg, 200 mg, and placebo groups, there was a mean change from baseline in BCVA of 0.2 ± 4.1, − 0.9 ± 6.6, 1.7 ± 5.6, and − 0.3 ± 10.6 letters at week 52, respectively. 6.7, 6.0, 4.7, and 8.1 rescue intravitreal injections were needed in the 50 mg, 100 mg, 200 mg, and placebo groups, respectively. The trial was prematurely stopped after the second interim analysis owing to gastrointestinal and hepatobiliary AEs, including diarrhea, vomiting, and fatigue, which occurred in a dose-dependent response to vorolanib. Transaminase elevation was also observed in this study, and alanine aminotransferase (ALT) and aspartate aminotransferase (AST) levels increased in 23.1% of the patients in the 200 mg group (Table 4) [98]. Another phase I clinical study to evaluate the safety and efficacy of oral vorolanib was conducted by AnewPharma in China, but the trial was suspended since 80.5% of the participants experienced TAEs. In the 75 mg group, elevated ALT, elevated AST, elevated blood pressure, leukopenia, and thrombocytopenia occurred in 62.5%, 50.0%, 37.5%, 37.5%, and 37.5% of participants, respectively (Table 4) [136]. The completion of a phase II single-arm study (NCT03710863) by AnewPharma was expected for January 2022; however, the status of the trial is still unknown (Table 4) [137].

#### EYP-1901

The potential of vorolanib to fight ocular neovascularization has been recognized by other pharmaceutical companies as well. Therefore, this molecule was also formulated into a bioerodible intravitreal implant (Durasert®) to treat nAMD and DR. Through intravitreal administration of EYP-1901 (DURAVYU®; EyePoint Pharmaceutical; Watertown, MA, USA), systemic AEs observed in clinical studies with oral vorolanib are assumed to be avoided. The product is designed for intravitreal administration twice a year, which is less frequent than in the case of anti-VEGF agents; however, non-serious OAEs can still be expected.

DAVIO2 was planned to be a randomized, active-controlled, phase II trial investigating EYP-1901 in patients with nAMD. In December 2023, EyePoint Pharmaceutical announced that DAVIO2 had met its primary endpoint. Both the 2 mg and 3 mg doses of EYP-1901 demonstrated a statistically non-significant change in BCVA compared with 2 mg aflibercept at week 32, thereby providing proof of non-inferiority. Mean gains from baseline were 1.0 and 0.9 letters in the 2 mg and 3 mg EYP-1901 groups, respectively, compared with the 1.3 letter gain in the aflibercept group. No treatment-related SOAEs or SSAEs were reported. Nonetheless, the rates of some OAEs were higher in the EYP-1901 arms than in the aflibercept arm (Table 4) [138]. Two additional ongoing phase II clinical studies are testing EYP-1901, developed by EyePoint Pharmaceutical. In February 2025, the company announced positive 6-month results for the randomized, active-controlled, phase II VERONA trial testing EYP-1901 in patients with DME. The 2.7 mg of EYP-1901 group demonstrated a 7.1 letter improvement in BCVA compared to baseline (Table 4). There were no treatment-related OAEs or SSAEs [139]. Furthermore, the company has announced the topline results of the phase II PAVIA clinical trial evaluating EYP-1901 as a potential nine-month treatment for NPDR. The trial did not meet the pre-specified primary endpoint (Table 4) [140].

#### CLS-AX

Axitinib is another TKI that inhibits all isoforms of VEGFR and, with a lower potency, the members of the PDGFR family (Fig. 1) [141]. CLS-AX (Clearside Biomedical; Alpharetta, GA, USA) is a suprachoroidal injection of axitinib that is being developed as a potential long-acting therapy for nAMD [142].

Recently, a phase I/IIa clinical study, OASIS, was conducted with positive safety outcomes in nAMD patients, and the phase II/b ODYSSEY trial achieved its primary and secondary outcomes. The company has announced that the analysis showed stabilization of patients’ BCVA (Table 4) [143, 144].

#### OTX-TKI

OTX-TKI (AXPAXLI®, Ocular Therapeutix; Bedford, MA, USA) is a bioresorbable, hydrogel-based implant of axitinib that can be administered intravitreally and is designed to treat nAMD and potentially DR by targeting VEGFRs.

The company announced that the phase I trial in nAMD had been completed, and more than 300 patients have been randomized in the SOL-1 phase III trial in December 2024. The company is planning to report topline data in the fourth quarter of 2025 (Table 4) [145, 146].

#### Nintedanib

Nintedanib is an oral TKI that targets a variety of growth factor receptors, including VEGFRs, PDGFRs, and FGFRs (Fig. 1) [147]. It is primarily indicated for the treatment of idiopathic pulmonary fibrosis.

Although nintedanib has not yet been tested in human subjects in ophthalmological indications, it has shown promising efficacy in a laser-induced CNV mouse model. The authors of the study found that nintedanib attenuated the development of CNV [148]. In a recent study, nintedanib was also investigated in vitro and in vivo using a streptozotocin-induced DR mouse model. They found that this TKI suppressed migration, tube formation, and proliferation of human retinal microvascular endothelial cells in vitro. Furthermore, it reduced the phosphorylation of VEGFR-2, FGFR-1, and PDGFR-β in vivo [122].

#### Sorafenib

Finally, sorafenib is also a multireceptor TKI inhibitor that inhibits VEGFRs and PDGFRs (Fig. 1) [149]. There are two published case studies in which ranibizumab was combined with orally administered sorafenib in two patients with nAMD, since both wanted to decrease the number of intraocular injections. In the first case, the patient received intravitreal ranibizumab and concomitantly 200 mg of oral sorafenib three times a week for 5 weeks. During the 5 weeks, the patient’s vision improved from 20/70 to 20/60. One month after the patient discontinued sorafenib therapy, his vision decreased back to 20/70. Subsequently, the patient started using oral sorafenib alone, and his vision improved to 20/50. In the second case, the patient’s VA was 20/30 in the left and 20/20 in the right eye, and previously received 8 ranibizumab injections. He opted to take 200 mg of oral sorafenib three times a week for a month, and his VA remained stable [124].

### Comparison of VEGF-targeting biotechnological products applied at present via meta-analysis of human clinical trials

#### Comparing treatment efficiencies

**Fig. 4:**
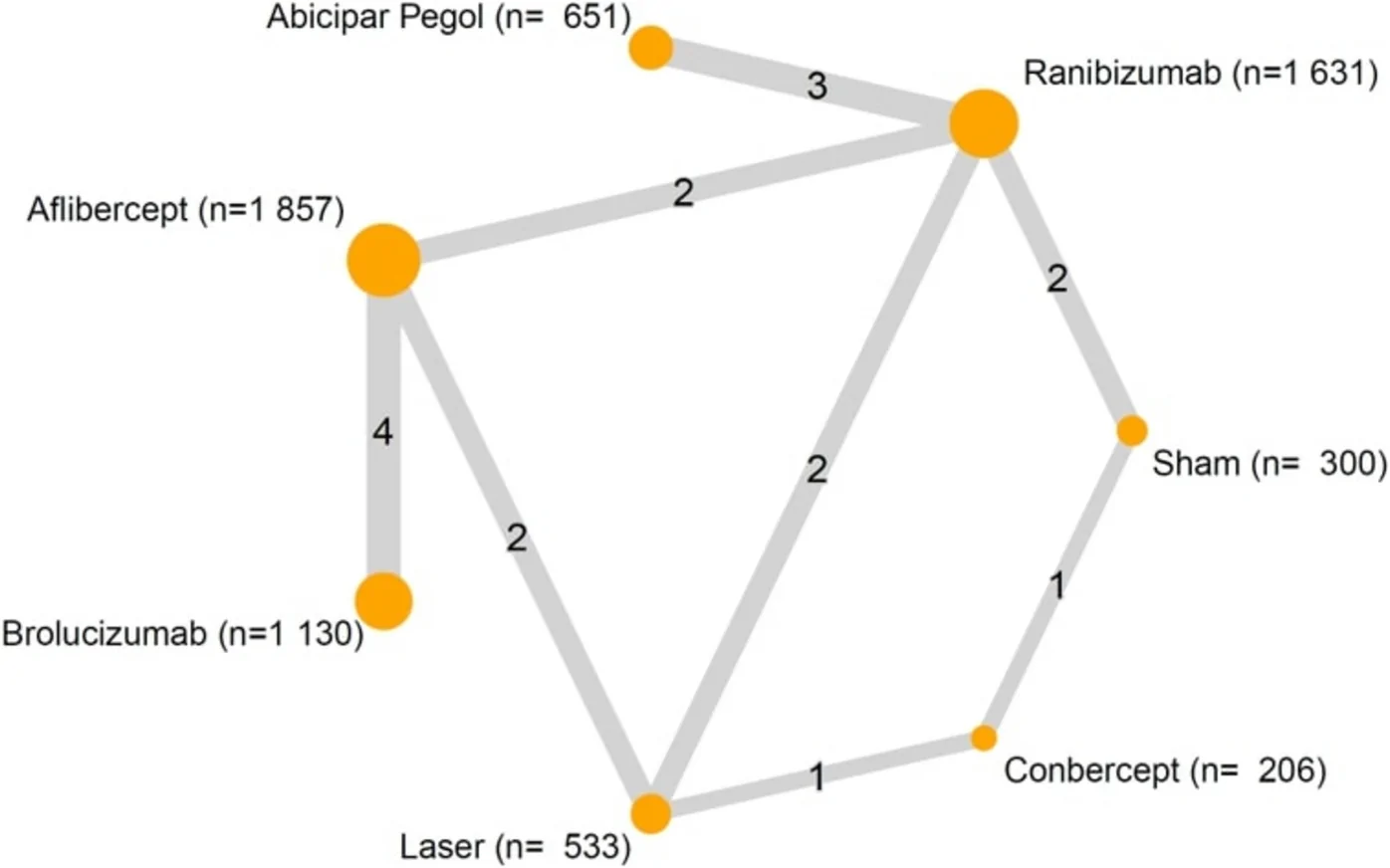
Network plot of anti-VEGF studies that assessed efficacy outcomes based on best-corrected visual acuity (BCVA). The size of the node and the thickness of the straight line are proportional to the number of trials and the presence of direct evidence between compared agents

**Fig. 5:**
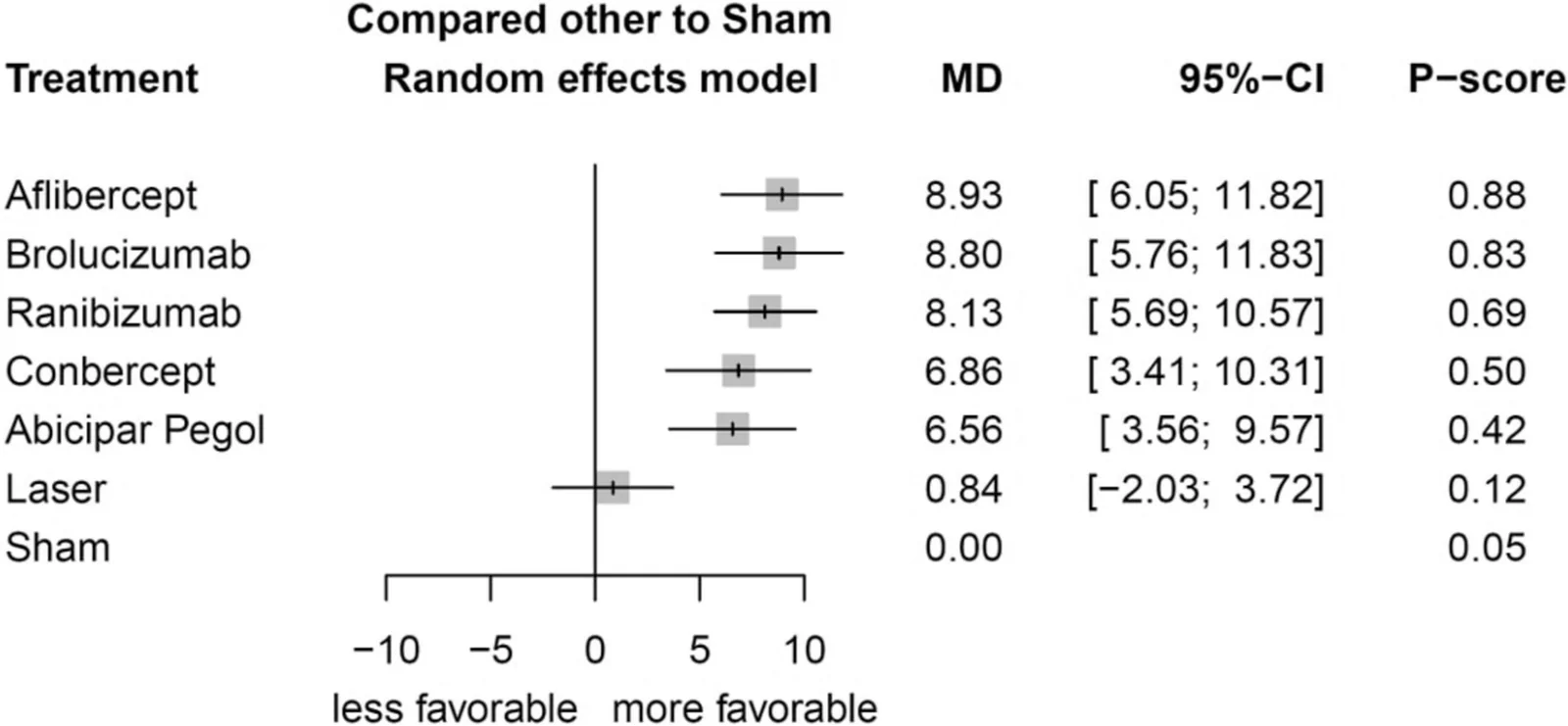
Mean difference of efficacy of anti-VEGF agents compared with sham. Based on mean BCVA change from baseline

A total of 17 studies involving 7 distinct treatment arms and 6308 participants were included in the network meta-analysis. Sham served as the reference group across all comparisons (Fig. 4). Using a random-effects model with the inverse variance approach, statistically significant differences in mean outcomes in BCVA were observed for several treatments when compared to the sham. Specifically, abicipar pegol, aflibercept, brolucizumab, conbercept, and ranibizumab demonstrated superior efficacy relative to the reference group (Fig. 5).

The ranking of treatments, determined by the P-score method, further highlighted variations in their relative effectiveness. Among the therapies, aflibercept emerged as the most effective intervention, achieving the highest P-score of 0.88. This score indicates a strong likelihood of aflibercept’s being among the top-performing treatments. In contrast, laser was consistently the least effective, with a P-score of 0.12, reflecting its position at the bottom of the efficacy hierarchy.

**Fig. 6:**
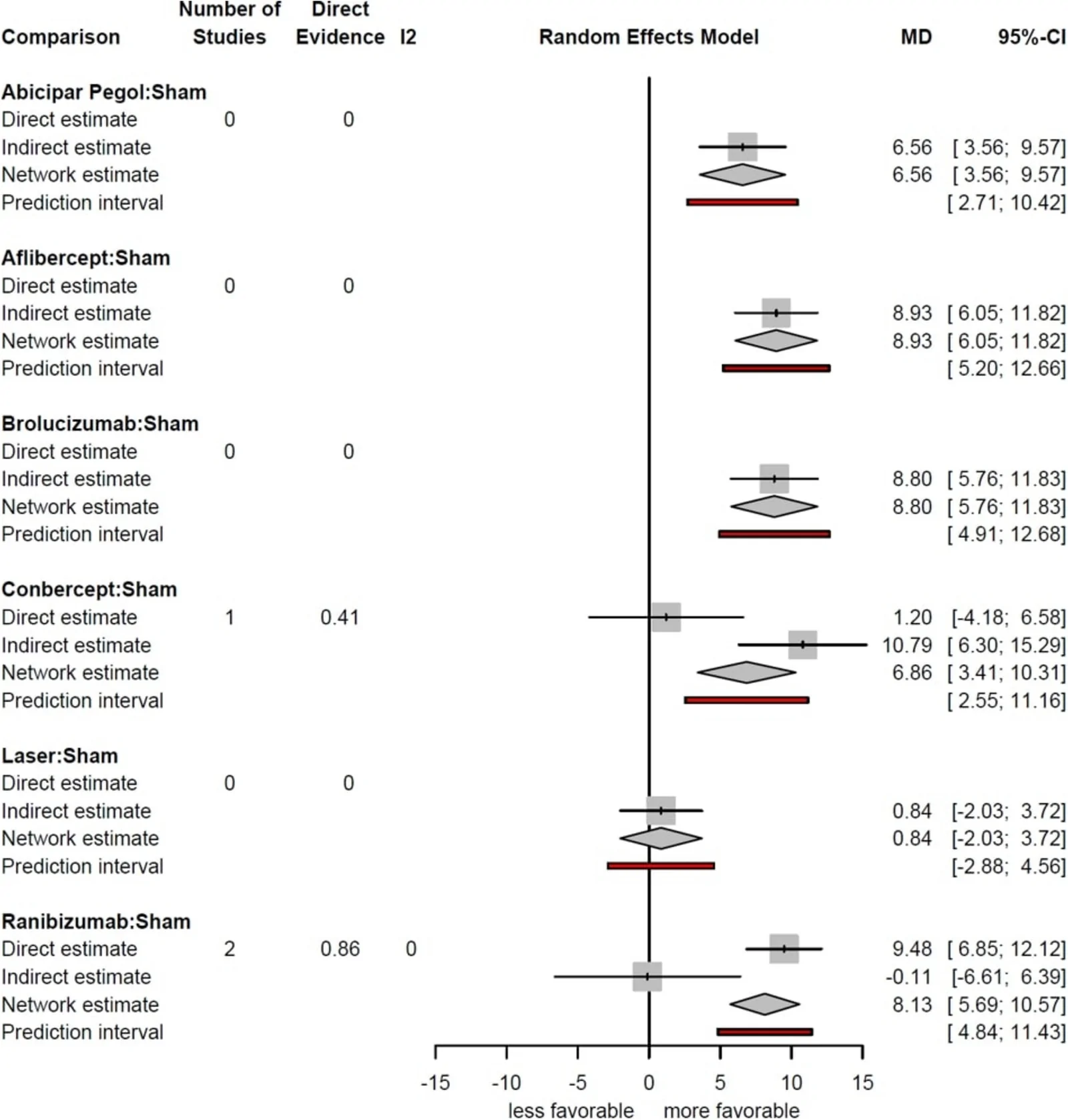
Assessment of direct and indirect evidence from treatment comparisons

The analysis also assessed the consistency of direct and indirect evidence across treatment comparisons. Statistically significant inconsistencies were observed in two treatment pairs: conbercept versus sham and ranibizumab versus sham. For both pairs, the *p*-values were 0.02 and 0.007, respectively, indicating that the direct and indirect estimates of treatment effects were not in agreement (Fig. 6). This suggests that caution may be needed when interpreting the results for these specific comparisons, as the observed discrepancies could reflect underlying complexities in the data or differences in the methodologies used to generate direct and indirect evidence.

These findings suggest that all active treatments examined provide substantial benefits over sham, with aflibercept standing out as the most promising option in this network.

#### Comparing treatment adverse events

**Fig. 7:**
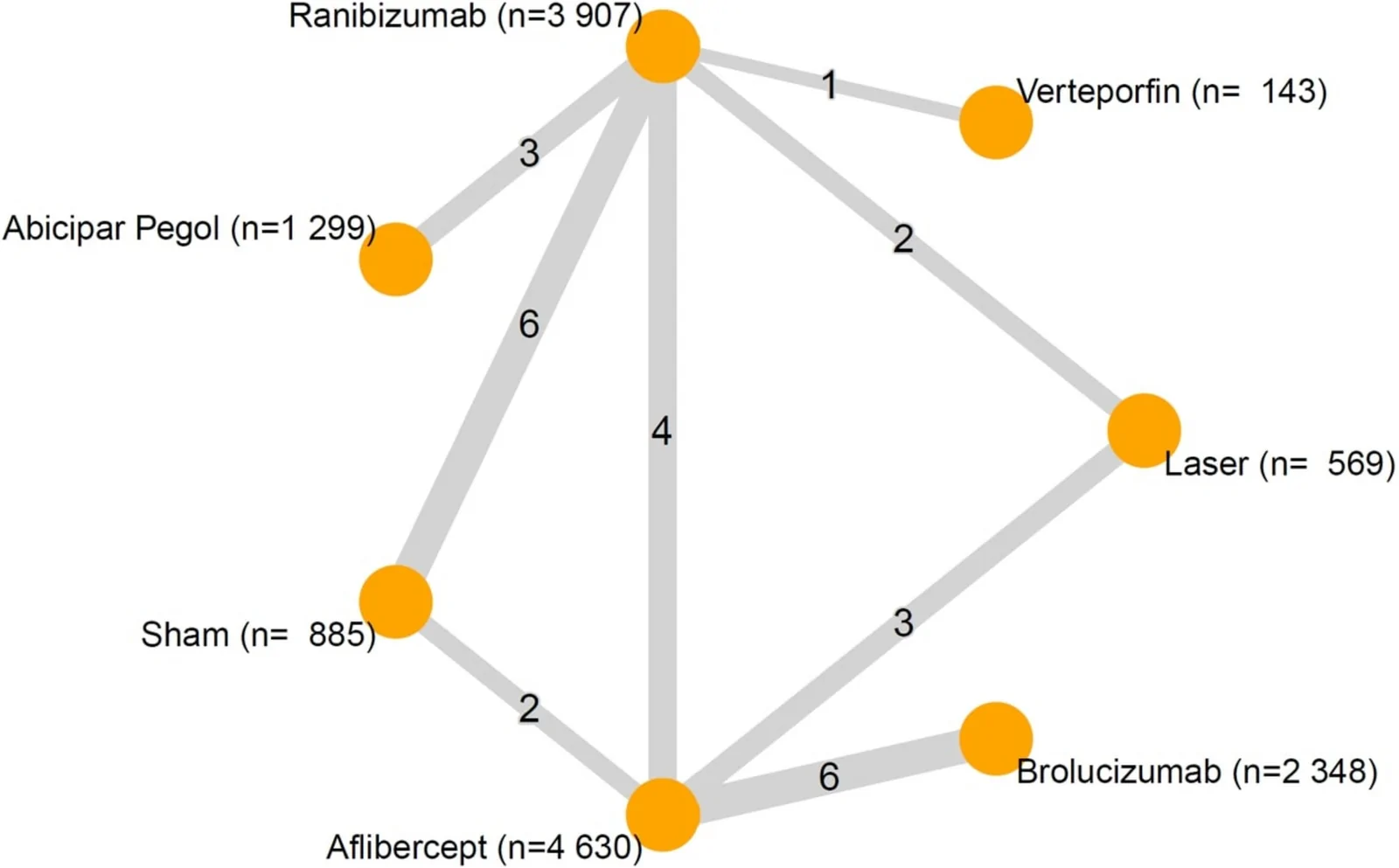
Network plot of anti-VEGF studies that assessed safety outcomes based on serious endophthalmitis events. The size of the node and the thickness of the straight line are proportional to the number of trials and the presence of direct evidence between compared agents

A total of 27 studies comprising 13,781 participants and seven treatment arms were analyzed. Sham served as the reference group, and no significant inconsistency was detected across the studies (Fig. 7).

**Fig. 8:**
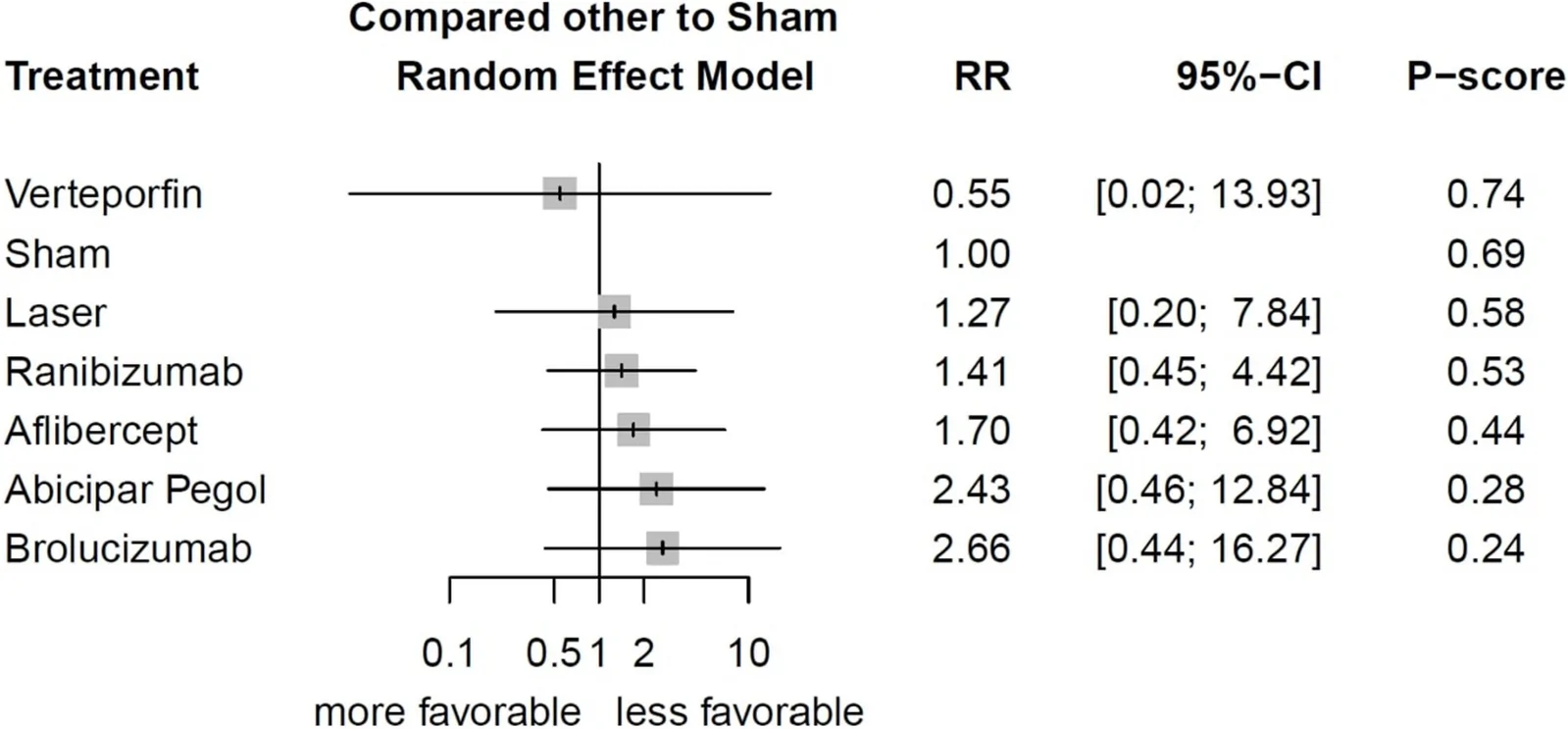
Risk ratio of serious endophthalmitis caused by anti-VEGF agents compared with sham. Based on serious endophthalmitis events

The analysis using a random-effects model and inverse variance method demonstrated no statistically significant difference in the risk ratio of serious endophthalmitis between any treatment and the control group (Fig. 8). Treatment ranking based on the P-score method indicated that verteporfin had the most favorable profile (P-score 0.74), while brolucizumab ranked lowest (P-score 0.24). Pairwise comparisons showed no statistically significant differences in risk between treatments (Table 5).

**Table 5:**
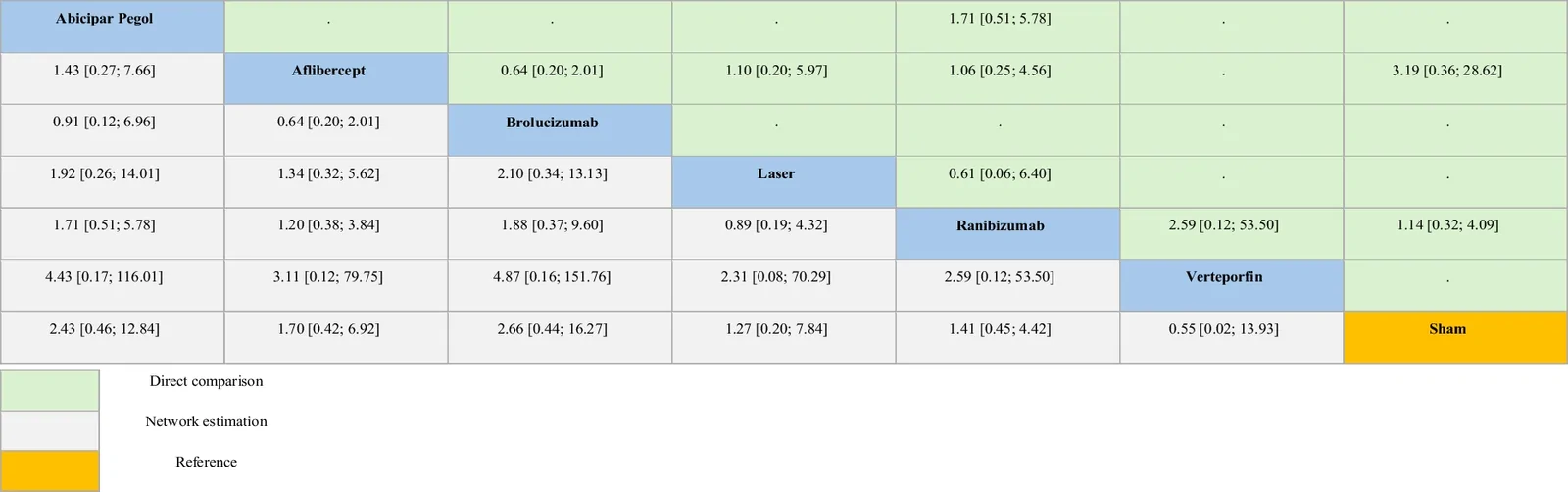
Pairwise comparison of the risk of serious endophthalmitis between treatments

## Conclusions

Currently, nAMD and PDR are the leading causes of vision loss in developed countries [1, 2]. VEGF family members, including VEGF-A, -B, -C, -D, PlGF, and PDGF, are important for the regulation of physiological vasculogenesis and angiogenesis but are also key factors in the deterioration of vision of nAMD and PDR patients [19]. The ethiology of these ophthalmological conditions includes pathological angiogenesis, which is the result of an imbalance between pro- and anti-angiogenic factors.

The purpose of this work was to review the efficacy of first-ever treatments used in the past for nAMD and PDR, the currently applied approaches, and novel promising therapies. The past 40 years have seen increasingly rapid advances in the field of treating ocular neovascularization, and more can be expected in the future. The data we have extracted from clinical studies underscore the concept that currently available approaches, including anti-VEGF therapy based on biotechnological products, are much more effective than laser photocoagulation and PDT [29–40, 53–57, 61, 62, 66–68, 71, 72, 150–154]. However, inspection of the most recent and ongoing clinical studies also forecast that gene therapy, and especially small-molecule TKI-based treatment, can further advance the field. Hence, these novel approaches can further ease the burden that afflicts patients with neovascularization [96, 100, 106, 107, 132–134, 136, 139, 140, 142, 144].

Regarding the currently applied gold standard anti-VEGF therapy, enough human clinical data have been accumulated so that we could conduct a frequentist network meta-analysis. According to the results of this approach, aflibercept was the most effective intervention compared with sham, followed by brolucizumab and ranibizumab. These results are coherent with the fact that aflibercept is the most frequently prescribed anti-VEGF therapy. On the other hand, laser photocoagulation emerged as the least effective treatment compared with anti-VEGF agents; therefore, our meta-analysis quantitatively justified phasing this method out during the last decades. Taken together, our results provided a coherent comparison of the different anti-VEGF therapies in terms of efficacy.

Despite their effectiveness, attention has to be paid to the less and more serious AEs occurring during anti-VEGF therapy. In ophthalmological indications, to avoid systemic AEs, one of the solutions is targeting anti-VEGF agents into the eye. However, the frequency of SOAEs such as endophthalmitis, retinal detachment, hemorrhages, and elevated intraocular pressure associated with repeated intravitreal injections constitutes an important and well-described burden of this approach. In our meta-analysis, we compared the risk ratio of serious endophthalmitis between anti-VEGF agents and the sham group. Brolucizumab, abicipar pegol, and aflibercept had the least favorable profile between the different treatment groups; however, the results have demonstrated no statistically significant difference between any of the groups. These results contradict the idea that endophthalmitis is a real disease-related problem that has a major impact on patients treated with anti-VEGF therapy. Presumably, clinical trials are conducted under more rigorous control of sterility, the lack of which is the main cause of endophthalmitis in everyday medical practice. Taking into account that intravitreal injections pose a risk of endophthalmitis and other SOAEs, our results warrant further human clinical studies, and preferentially retrospective ones on an extended patient population. Moreover, even though being administered intravitreally, anti-VEGF agents could leak into the plasma. Correspondingly, they can cause SSAEs as well, including arteriothrombotic events by inhibiting VEGF in the circulation [58–60].

To reduce the number of annually applied anti-VEGF injections, several gene therapy products are under clinical development to treat nAMD and PDR. According to clinical trials, these products show relatively good efficacy; however, given the intravitreal, suprachoroidal, or subretinal administration route, various OAEs, including cataract, retinal detachment, and uveitis, are still observed during the treatment [96, 108]. To date, there is limited data available from completed clinical trials. Therefore, we currently do not know the long-term side effects caused by gene therapy. One of the possible serious AEs of the treatment is gene silencing and insertional mutagenesis, which can cause tumor formation [92, 93]. Gene therapy products, which are currently being tested to target nAMD and PDR, are based on AAV vectors. Although the recombinant AAV vectors primarily remain in the episome, some mouse studies suggest that these vectors can also cause insertional mutagenesis, which has been linked to hepatocellular carcinomas in mice [155, 156]. This shows that all vectors have integration risk, and the frequency depends on the type of vector. According to current guidance by the FDA and EMA for study sponsors, companies developing gene therapy products should enroll patients who have received gene therapy in a long-term follow-up safety study. During the 5–15-year period, they should continue to assess the safety of their developed gene therapy products to understand the risk of delayed adverse events associated with the integration activity and the genome editing activity of the gene therapy product and the long-term effects of the prolonged expression of the therapeutic protein. Some studies have also shown that AAV vector-based gene therapy can cause elevated levels of AST and ALT, resulting in hepatotoxicity [157, 158]. Another concern regarding gene therapy is its affordability. Some of the approved gene therapies are among the most expensive medicines, which are usually not covered by health insurance. This makes them hardly accessible to patients, especially in developing countries. All things considered, if the serious side effects and long-term risks could be prevented, a prolonged VEGF/VEGFR inhibition can be achieved with the currently developed gene therapies against ocular neovascularization.

Recently, small-molecule TKIs have been proposed for the treatment of retinopathies, and the promising results of completed and currently ongoing clinical studies are already available. A huge benefit of TKIs compared to anti-VEGF treatment and gene therapy is that they can be administered orally, thus significantly increasing patient compliance. Importantly, OAEs can be completely avoided. However, systemic AEs can be observed during treatments with TKIs; therefore, not all of them are suitable for ophthalmological indications [97, 98, 136, 137]. According to various studies, TKIs show promising efficacy in angiogenesis inhibition. Despite this observation, none of the therapies based on TKIs are approved in Europe or the USA. The plausible reason behind this is that oral TKIs are associated with gastrointestinal AEs and hepatotoxicity, while depot formulations and implants of TKIs increase the rates of OAEs [132–134]. Recently, to minimize the systemic risk factors, there have been attempts to investigate novel routes of drug delivery. These recent approaches include topical delivery of several TKIs to the retina or the formulation of a TKI into cyclodextrin nanoparticles delivered via the same route [159, 160].

Vorolanib has been developed using the sunitinib scaffold to decrease the AEs and increase the specificity towards the VEGFR and PDGFR family. Although oral vorolanib has failed in clinical trials for ophthalmological indications because of the still intolerable hepatotoxicity, an intravitreal formulation of vorolanib (EYP-1901) successfully passed phase I and is currently being investigated in phase II for three different ocular indications [97, 98, 136, 138–140]. Accordingly, a promising approach would be to further modify the structure of vorolanib or other already existing TKIs while maintaining the same strength of inhibition towards the VEGFR and PDGFR family [161]. In this way, novel, less-toxic molecules could be engineered. Future research should, therefore, concentrate on the investigation of such promising TKIs to create safe, long-term therapy to prevent blindness caused by nAMD and PDR.

Taken together, the primary benefit of gene therapy products and TKIs is to enhance patient compliance and to reduce ocular infections. From this point of view, they appear to be promising future treatments for nAMD and PDR, but more improvements are needed to meet the expected level of efficacy, which is set by routinely applied anti-VEGF therapies. Therefore, future developments should focus as much on improving the efficacy as on improving the safety profile of these products.

## Data Availability

The data used and analyzed in this study are available from the corresponding author upon reasonable request.

## Abbreviations

AAV: Adeno-associated virus
AEs: Adverse events
ALT: Alanine aminotransferase
AMD: Age-related macular degeneration
Ang2: Angiopoietin-2
AST: Aspartate aminotransferase
BCVA: Best-corrected visual acuity
CAGR: Compound annual growth rate
CATT: Comparison of Age-related Macular Degeneration Treatments Trials
c-Kit: Stem cell factor receptor
CNV: Choroidal neovascularization
dAMD: Dry age-related macular degeneration
DME: Diabetic macular edema
DR: Diabetic retinopathy
DRS: Diabetic Retinopathy Study
ETDRS: Early Treatment Diabetic Retinopathy Study
FGF-2: Fibroblast growth factor-2
FGFR: Fibroblast growth factor receptor
Flt-3: Fetal liver tyrosine kinase 3
HIF-1α: Hypoxia-inducible factor 1α
ICD: Intracellular domain
Ig: Immunoglobulin
IOI: Intraocular inflammation
MAC: Membrane attack complex
nAMD: Neovascular age-related macular degeneration
NPDR: Non-proliferative diabetic retinopathy
OAEs: Ocular adverse events
PDGF: Platelet-derived growth factor
PDGFR-β: Platelet-derived growth factor receptor β
PDR: Proliferative diabetic retinopathy
PDT: Photodynamic therapy
PlGF: Placental growth factor
PRP: Panretinal photocoagulation
ROS: Reactive oxygen species
RPE: Retinal pigment epithelium
SOAEs: Serious ocular adverse events
SSAEs: Serious systemic adverse events
T&E: Early-start treat-and-extend
TAEs: Treatment-related adverse events
TAP: Treatment of AMD with Photodynamic Therapy
TKIs: Tyrosine kinase inhibitors
VA: Visual acuity
VEGF: Vascular endothelial growth factor
VEGFR: Vascular endothelial growth factor receptor
VPF: Vascular permeability factor
